# A nutrient control on oxygenation dynamics during Earth’s Great Oxidation Episode

**DOI:** 10.1038/s41467-026-76597-y

**Published:** 2026-08-27

**Authors:** Lewis J. Alcott, Benjamin J. W. Mills, Andrey Bekker, Zidong Peng, Simon W. Poulton

**Affiliations:** 1 School of Earth Sciences, University of Bristol, Bristol, UK; 2 School of Earth, Environment and Sustainability, University of Leeds, Leeds, UK; 3 Department of Earth and Planetary Sciences, University of California, Riverside, CA USA; 4 Department of Geology, University of Johannesburg, Johannesburg, South Africa; 5 Institute of Mineral Resources, Chinese Academy of Geological Sciences, Beijing, China

**Keywords:** Element cycles, Geochemistry

## Abstract

Earth’s first rise in atmospheric oxygen occurred over a protracted period from ca. 2.43 to 2.06 billion years ago during the Great Oxidation Episode (GOE). The GOE was marked by extreme climatic instability, which likely impacted the bioavailability of the principal limiting nutrient, phosphorus. Here, we investigate phosphorus cycling across the final two glaciations of the GOE, as documented by the Transvaal Supergroup, South Africa. Utilising a phosphorus phase partitioning approach, we show that following an enhanced weathering-induced phosphorus influx that stimulated oxygenation in the aftermath of glaciation, sedimentary phosphorus recycling was controlled by feedback-driven fluctuations in sulphate availability and oceanic redox state. Biogeochemical modelling suggests that this dynamic interplay drove major instability in atmospheric oxygen levels during Earth’s protracted transition to a persistently oxygenated atmosphere.

## Abstract (web summary)

Earth’s first rise in atmospheric oxygen was marked by extreme climatic instability. Phosphorus phase partitioning and biogeochemical modelling show that feedbacks between nutrient availability, redox and deglaciation controlled oxygenation.

## Introduction

The Great Oxidation Episode (GOE) documents the transition to a permanently oxygenated atmosphere^1^, and represents a defining interval in Earth history with respect to the subsequent evolution of seawater redox chemistry and the role of marine oxygenation in the course of biological evolution^2–4^. While multiple independent lines of evidence support an overall rise in atmospheric oxygen across the GOE^4^, the loss of mass-independent fractionation of sulphur isotopes (MIF-S) provides the most precise tool for assessing the timing and dynamics of atmospheric oxygenation^1,5–12^. Indeed, based on improved, geochronologically calibrated multiple sulphur isotope records, the onset of the GOE has been constrained to ca. 2.43 billion years ago (Ga)^6,7^, which represents the initial rise of atmospheric oxygen to above 10^−6^ PAL (Present Atmospheric Level)^13,14^.

The overall transition to higher atmospheric oxygen levels is linked to extreme perturbations in global climate^1,6,15^. Global lithostratigraphic correlations infer multiple glacial intervals during the GOE, with the widespread, low-latitude extent of earlier glaciations implying possible ‘Snowball Earth’ conditions, prior to the final, more regional glaciation at ca. 2.25 Ga^6,16–18^. A strong interplay between these glacial events and fluctuating oxygen levels is apparent, with oxygenation peaking during non-glacial intervals^1,6,9,19,20^. While this oscillatory behaviour likely occurred on a global scale, possible spatial heterogeneity in the MIF-S signal highlights that its manifestation in the marine geological record is complex^11,12^. Going further, the recognition of oscillatory oxygen levels from MIF-S signals is further complicated by potential detrital contributions and post-depositional overprints^21,22^. Nevertheless, recent work has demonstrated that the aftermath of the final widespread glaciation marks the onset of coupled atmosphere-ocean oxygenation^23^, as indicated by a clear coupling between lower thallium isotope ratios (documenting ocean oxygenation) and the disappearance of MIF-S (documenting atmospheric oxygenation)^1^.

While the precise nature of oceanic redox conditions and their dynamics across the GOE remains poorly resolved, it is clear that deeper waters remained predominantly ferruginous (anoxic and Fe^2+^-bearing)^2^, with spatially and temporally restricted euxinia (anoxic and sulphidic water-column conditions) occurring in shallow waters, at least in the aftermath of the final widespread glaciation^1^. These redox dynamics would have greatly impacted the bioavailability of phosphorus (P), the ultimate limiting nutrient on geological timescales^24^, which is thought to have been fundamental in priming and driving the GOE^25,26^.

Under ferruginous conditions, there is potential for extensive drawdown and fixation of P through uptake in association with iron minerals^27,28^. However, sulphate and organic carbon availability may diminish this iron trap through the production of H_2_S during early diagenesis, leading to the reductive dissolution of iron (oxyhydr)oxide minerals and release of associated P^25,29,30^. This release of P to pore-waters and seawater is commonly enhanced by the preferential release of organic-bound P (P_org_) relative to organic carbon (C_org_) during anaerobic remineralization, leading to elevated molar C_org_/P_org_ ratios relative to the Redfield ratio of 106:1^31,32^. After release into sediment pore-waters, P may be fixed via ‘sink-switching’ to authigenic phases, such as carbonate fluorapatite (CFA)^33,34^ or vivianite^34–36^. However, under anoxic water-column conditions, particularly when the water column is euxinic, or when pore-waters are sulphidic close to the sediment-water interface (which removes the Fe-(oxyhydr)oxide trap for P), P can be extensively recycled back to the water column, where it may stimulate further productivity, leading to a net increase in oxygen production^25,30,37^. However, these redox-promoted biogeochemical processes have not been investigated for any part of the GOE, and hence nutrient controls on dynamic atmospheric oxygen levels across the GOE remain entirely unconstrained.

To investigate the role of P cycling in driving oxygenation dynamics across the GOE, we focus on shale samples in well-preserved drill-core from the ca. 2.32-2.25 Ga Rooihoogte and Timeball Hill formations of the Transvaal Supergroup, South Africa. This succession documents clear atmosphere-ocean redox changes across the final widespread glaciation and subsequent regional glaciation of the GOE^1,23,38^, and we build upon existing redox data with new redox-sensitive trace element (RSTE) analyses, alongside a detailed study of P phase partitioning. In combination with biogeochemical modelling, this approach demonstrates how feedbacks between P cycling, oceanic redox conditions and climate drove major instability in oxygenation during Earth’s protracted transition to a persistently oxygenated atmosphere.

## Results and discussion

### Atmospheric and oceanic redox conditions

**Fig. 1:**
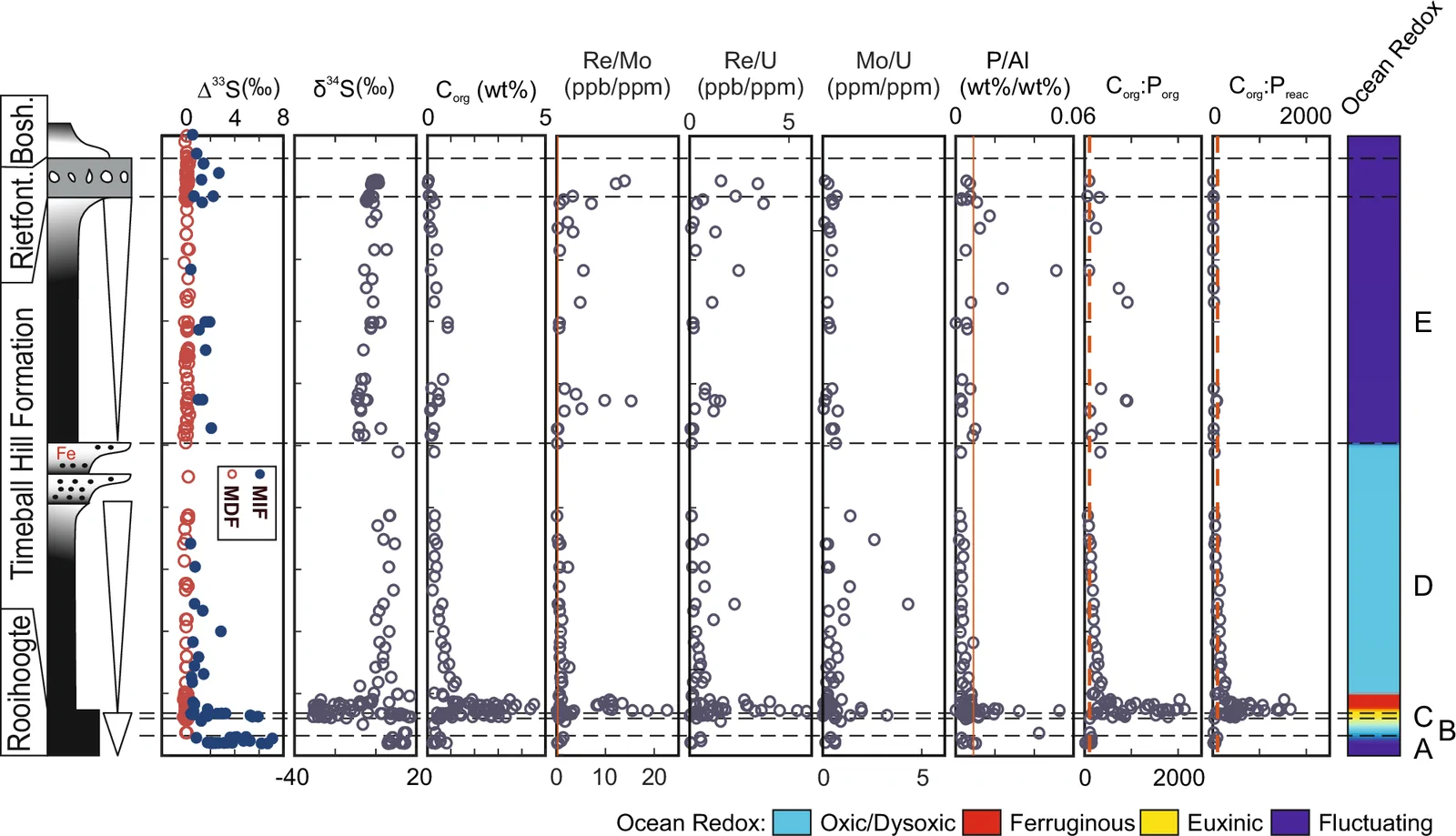
Geochemical and isotopic profiles through drill-core EBA-2. Trends in atmospheric oxygenation and ocean redox conditions are based on Fe-S^1^ and redox-sensitive trace elements (this study) systematics. Red lines on U/Al, Mo/Al and Re/Al plots represent average ratios for upper continental crust^49^. Multiple sulphur isotope data^1^ are included to emphasise the atmospheric oxygenation history, and the Δ^33^S record also includes data from an additional drill core (EBA-1). Red lines on C_org_:P_org_ and C_org_/P_reac_ plots denote the Redfield ratio (106:1). C_org_ – organic carbon, P_org_ – organic bound phosphorus, P_reac_ - reactive phosphorus. Intervals (**A–E**) correspond to broad atmosphere-ocean redox intervals^1^, but we note that even higher-frequency redox fluctuations may have occurred within these broad intervals^12^. The third glacial interval of the Great Oxidation Episode (GOE) is older than the Rooihoogte Formation. Roo.- Rooihoogte Formation, Riet. – Reitfontein Diamictite in the Timeball Hill Formation, Bosh. - Boshoek Formation.

Our new RSTE (shown as Re/U, Re/Mo and Mo/U ratios^39^) and P phase partitioning data for drill-core EBA-2 (see Methods and Supplementary Information for a full description of the geological setting, analytical techniques and all data) are presented alongside existing multiple S isotope and C_org_ concentration data^1^ in Fig. 1. The Rooihoogte and Timeball Hill formations in drill-core EBA-2, which were deposited in a shallow-marine, deltaic setting with unrestricted connection to the ocean^40,41^, capture the nature of oxygenation in the aftermath of the final widespread glaciation, while the overlying Reitfontein Diamictite documents the subsequent regional-scale glaciation^42^ (Fig. 1).

Large MIF-S signatures indicative of a functionally anoxic atmosphere in the lower Rooihoogte Formation are initially interrupted by a zone of mass-dependent S isotope (MDF-S) values that have been suggested to reflect a transient interval of atmospheric oxygenation^1,10^, prior to a second apparent loss of the MIF-S signal across the Rooihoogte-Timeball Hill Formation boundary (Fig. 1). This second loss of the MIF-S signal has traditionally been considered to document the permanent rise of atmospheric oxygen^8,10^. However, the multiple S isotope record implies that fluctuations between a functionally anoxic and oxic atmosphere may have persisted through the overlying strata, with the permanent transition to an oxygenated atmosphere occurring in association with the Lomagundi C isotope excursion at ca. 2.22 Ga^1^. Nevertheless, while we consider the nature of P controls on atmospheric and ocean oxygenation throughout the section, our main focus is on the larger-scale ocean redox and nutrient interactions that occurred in the immediate aftermath of glacial melting, lower in the section.

Local, shallow-marine redox fluctuations through the section are closely linked to the dynamics of atmospheric oxygenation (Fig. 1; for simplicity, we divide the section into five intervals (A-E) based on the dominant atmospheric and oceanic redox state^1^). While our RSTE data are entirely consistent with redox reconstructions based on Fe-S systematics^1^ and lower-resolution RSTE (U and Mo) data^23^, our specific utilisation of RSTE ratios^39^ provides additional nuance due to the enhanced ability to distinguish dysoxic from oxic bottom-water conditions (see Supplementary Information Figs. S2–4). In brief, U sequestration commonly occurs under anoxic bottom-water conditions^43^, while Re is more sensitive to oxygen depletion and begins to be sequestered under dysoxic conditions^44,45^. By contrast, substantial drawdown of Mo either requires anoxia and the presence of dissolved sulphide, or a particulate shuttle mechanism whereby Mo (but not U or Re) is sequestered due to uptake by Fe minerals precipitating at the redoxcline above deeper ferruginous waters^46–48^. Therefore, high Re/U and Re/Mo values commonly indicate dysoxic conditions (further supported by high Re/Al and low highly reactive (Fe_HR_) to total Fe (Fe_T_) values), whereas low Re/Mo and high Re/U values commonly develop under anoxic, ferruginous conditions (supported by high Re/Al, U/Al and Fe_HR_/Fe_T_, and low pyrite (Fe_py_) to Fe_HR_ values)^37^. Euxinic conditions are commonly characterised by high Mo/U values, alongside high Fe_HR_/Fe_T_ and Fe_py_/Fe_HR_ values (also supported by high Re/Al, U/Al and Mo/Al, and low Re/Mo and Re/U values)^37^.

Utilising this framework alongside Fe-S systematics^1^ (Figs. S2, 3) provides evidence for fluctuating redox conditions under an anoxic atmosphere in interval A (Fig. 1). Specifically, oxic depositional conditions are indicated by low Re/U and Re/Mo values (Fig. 1), alongside generally low Fe_HR_^*^/Fe_T_ values (where Fe_HR_^*^ includes a correction for loss of Fe_HR_ to sheet-silicate minerals during diagenesis^1,2^; see Supplementary Information). Occasionally, elevated Fe_HR_^*^/Fe_T_ and Fe_T_/Al values do occur, however, with some samples having elevated Fe_py_/Fe_HR_ values (Figs. S2, 3). Nevertheless, Re/Mo and Re/U values remain low (Fig. 1), suggesting that Fe enrichments likely occurred due to periodic upwelling of ferruginous waters into oxic shallow-waters, with elevated Fe_py_/Fe_HR_ values likely reflecting diagenetic pyrite formation, which is consistent with relatively heavy δ^34^S pyrite values (Fig. 1). Thus, interval A appears to have experienced fluctuating seawater redox conditions linked to periodic upwelling.

In interval B, where multiple S-isotope systematics support atmospheric oxygenation^1,10^, the available ocean redox data are limited, but initially suggest oxic depositional conditions (Fig. S3; see also ref. ^1^ for supporting data from a second drill-core), followed by the development of euxinia towards the top of the interval (indicated by Fe-S systematics, elevated Mo/U ratios, and co-variation in Mo and U enrichment factors; Fig. 1 and Figs. S3, 4). Euxinia persisted as the atmosphere transitioned back to an anoxic state through interval C and into the basal part of the Timeball Hill Formation, before the development of ferruginous conditions (Fig. 1 and Fig. S3). This short-lived ferruginous interval brackets the initial stage of the second period of atmospheric oxygenation, before the atmosphere again transitioned to an anoxic state (Fig. 1).

Following the initial post-glacial redox dynamics, low Fe_HR_^*^/Fe_T_ ratios (Fig. S2), combined with generally low Re/U and Re/Mo ratios (Fig. 1), indicate predominantly oxic shallow-marine conditions through interval D, although the occurrence of occasionally elevated Re/U ratios suggests the periodic development of dysoxic conditions in bottom-waters or at the sediment-water interface^39^. Through interval E in the upper Timeball Hill Formation, fluctuations in Fe_HR_^*^/Fe_T_, Re/U and Re/Mo ratios (Fig. 1 and Fig. S2) indicate variability in water-column redox conditions (from ferruginous to dysoxic/oxic), prior to the development of more persistent ferruginous conditions leading up to, and during, the final regional glaciation (Fig. 1 and Fig. S2).

### Phosphorus dynamics

Phosphorus concentrations throughout much of the section (expressed as P/Al; Fig. 1) are commonly lower than upper continental crust values (0.009)^49^, with the exception of a notable zone of elevated values across the Rooihoogte-Timeball Hill formation boundary, and a few samples in the upper Timeball Hill Formation. These Rooihoogte-Timeball Hill formation boundary samples appear to document extensive P drawdown in association with organic matter (Fig. 1), highlighting a productivity control on P sequestration. However, the extent to which P is retained in the sediment during early diagenesis also affects the P loading of the sediments^31,32,50^. To assess this, we consider redox controls on C_org_/P_org_ and C_org_/P_reac_ ratios, where P_reac_ represents potentially bioavailable P (comprising organic, authigenic and Fe-bound P; see Supplementary Information).

We first note that it is not simply the dominant oceanic redox state that determines whether P is recycled back to the water column (indicated by C_org_:P_org_ and C_org_:P_reac_ ratios above 106:1) or retained in the sediment. Rather, it is the generation of dissolved sulphide during very early diagenesis that exerts a major control, which would itself have been affected (at least in part) by low (and fluctuating) sulphate concentrations prior to persistent oxygenation of the atmosphere^25^. As a first-order assessment of relative sulphate availability, we consider pyrite S isotope (δ^34^S) values (Fig. 1).

**Fig. 2:**
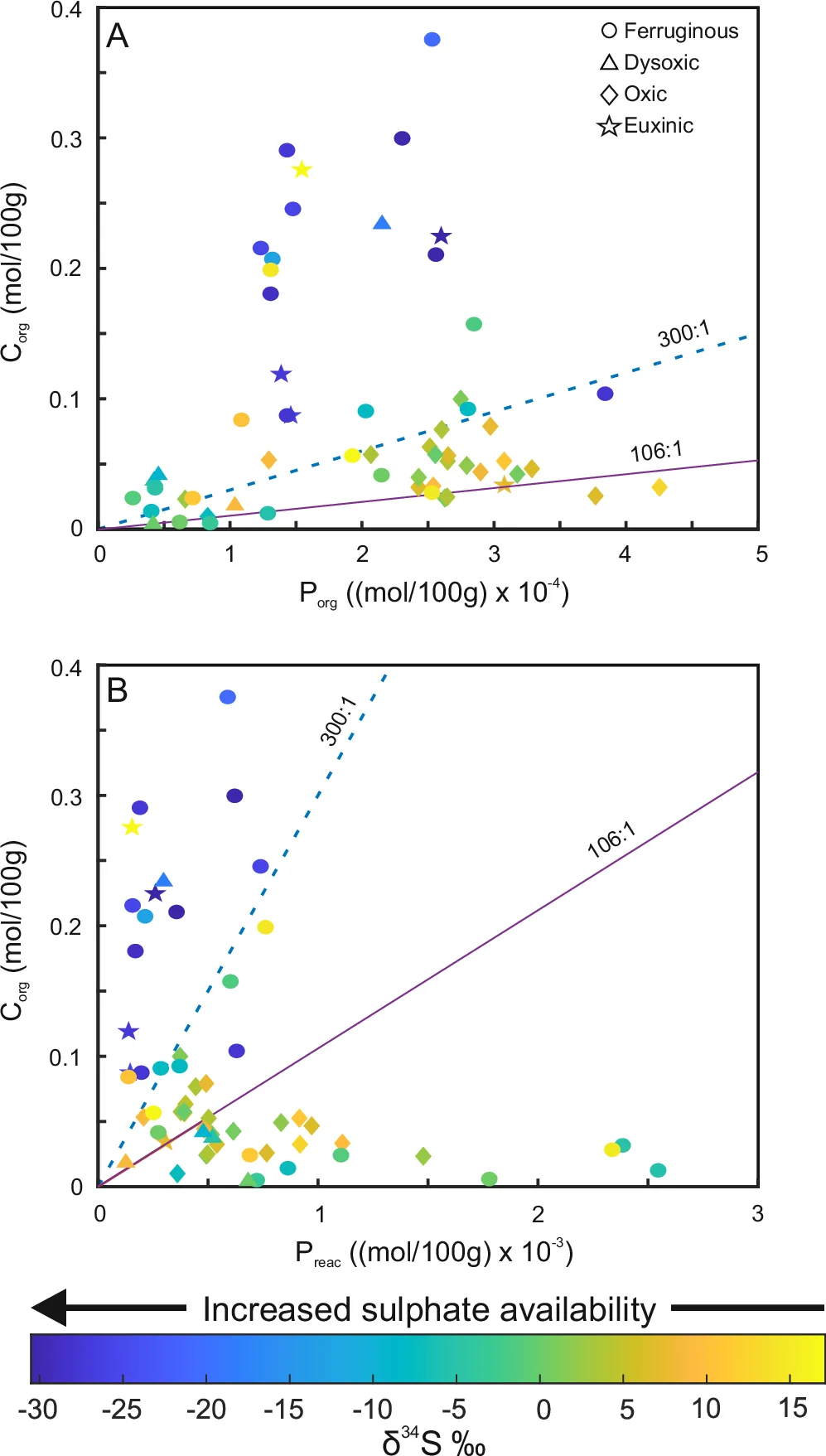
Relationship between organic carbon and different phosphorus pools for samples from drill-core EBA-2. **A** C_org_:P_org_; **B** C_org_:P_reac_. Black dashed lines represent the Redfield Ratio (106:1) and the blue dashed line represents an inferred C_org_:P_org_ ratio of 300:1 for early Precambrian sediments deposited under highly oligotrophic, P-limited conditions^27^. Symbols represent defined redox conditions as described. Colour of symbols denotes δ^34^S values. C_org_ – organic carbon, P_org_ – organic bound phosphorus, P_reac_ - reactive phosphorus.

Although there are a variety of potential controls on pyrite δ^34^S values in ancient sediments^4,51,52^, very low compositions (relative to the earlier Precambrian^4^) of about −35‰ across the Rooihoogte-Timeball Hill formation boundary (Fig. 1) are consistent with elevated sulphate concentrations (above 200 μM^53^). In considering alternative possibilities, we note that there is no sedimentological evidence for a change in sedimentation rate that might influence pyrite δ^34^S values^54^, and instead, the pyrite δ^34^S trends occur coincident with major changes in atmospheric oxygenation and ocean redox conditions (Fig. 1). This suggests an enhanced flux of sulphate to the oceans (which increases δ^34^S fractionation in pyrite^51,55^) driven by oxidative weathering of the continents linked to rising atmospheric oxygen^8^. We thus distinguish our P phase partitioning data based on the sulphur isotope composition, as a reflection of broad changes in seawater sulphate availability driven by differing intensity of oxidative continental weathering during fluctuating atmospheric oxygen levels and associated oceanic redox state (Fig. 2).

This approach clearly highlights that samples deposited during time intervals of probably increased seawater sulphate concentrations (dominantly corresponding to those at the Rooihoogte-Timeball Hill Formation boundary; Fig. 1) generally have higher C_org_/P_org_ and C_org_/P_reac_ ratios, regardless of the precise redox state of the water column (Fig. 2). The C_org_/P_org_ and C_org_/P_reac_ ratios for these samples are considerably elevated above both the Redfield ratio and an assumed ratio of 300:1^27^ for highly P-limited conditions in globally ferruginous Precambrian oceans (which clearly was not the case for these TOC-rich samples; Fig. 1), indicating extensive recycling of P back to the water column^31,56^. This group also includes ferruginous samples just above the Rooihoogte-Timeball Hill Formation boundary, where active microbial sulphate reduction is indicated by elevated Fe_py_/Fe_HR_ ratios (Figure S3), but not to the extent where euxinic bottom-waters developed (Fig. 1). By contrast, oxic, dysoxic and ferruginous samples deposited under probable lower seawater sulphate conditions commonly have C_org_/P_org_ ratios closer to the Redfield ratio, with C_org_/P_reac_ ratios that fall below this value (Fig. 2). This is consistent with minimal preferential release of P from buried organic matter during early diagenesis (consistent with relatively low Fe_py_/Fe_HR_ ratios for samples above the Rooihoogte-Timeball Hill formation boundary; Figure S2), coupled with enhanced drawdown of P in association with the precipitation of Fe minerals^25,28^.

### Climate and nutrient controls on oxygenation

**Fig. 3:**
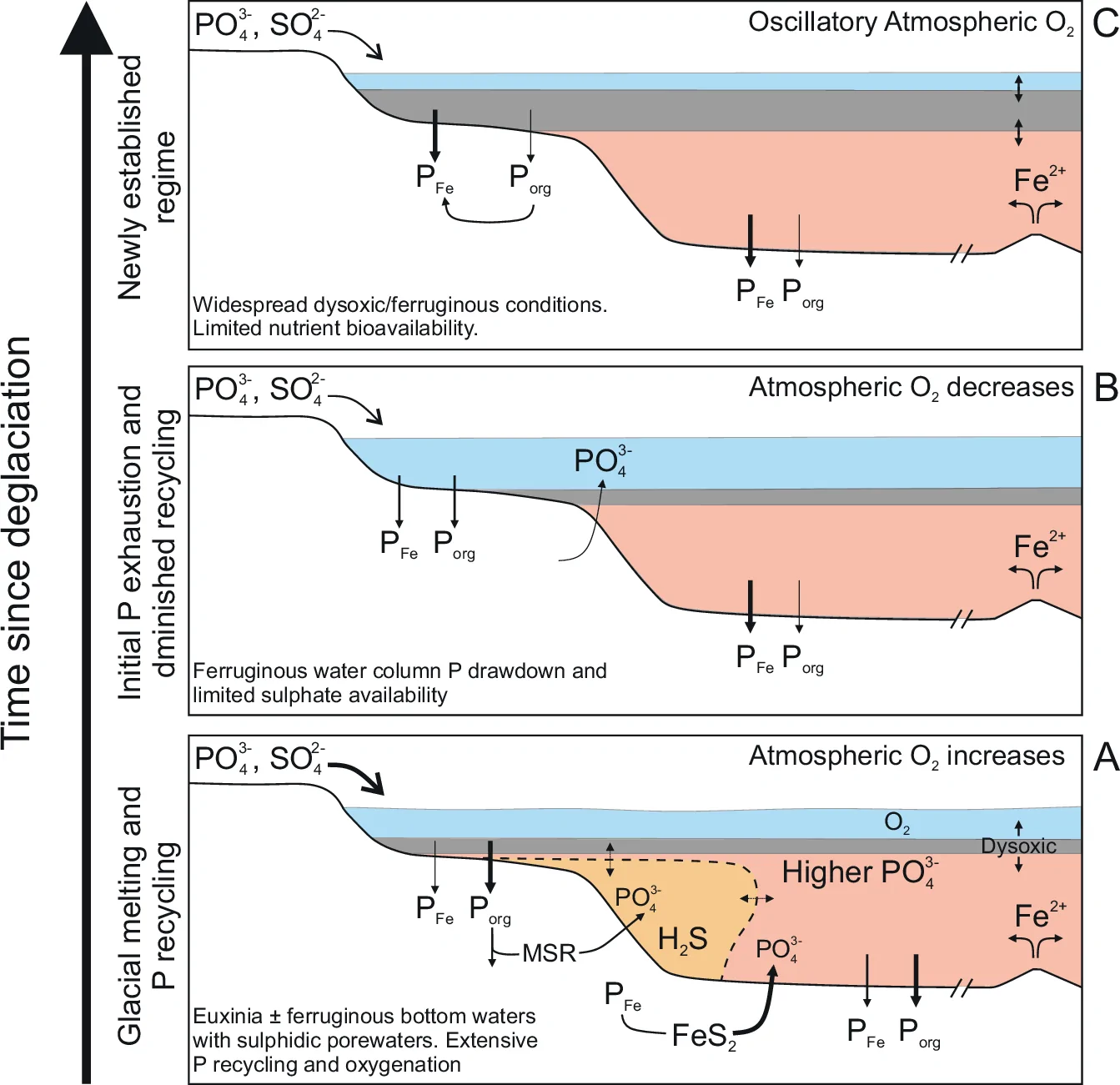
Illustrative model demonstrating the self-limiting feedbacks among the oxygen, sulphur and phosphorus cycles following glaciation. Panel progression from **(A–C)** denotes time since deglaciation, where **(A)** represents the post-glacial pulse of both phosphate and sulphate, which drove the initial rise of O_2_ through phosphorus recycling (intervals (A–C) in the text). **B** Demonstrates how feedbacks between phosphorus and sulphur led to progressive P exhaustion as euxinic conditions waned (interval (D) in the text). **C** Represents the new redox regime developed during deposition of the Timeball Hill Formation, whereby weathering fluxes returned to a new baseline and atmospheric oxygen level dropped from the peak values that occurred in the aftermath of deglaciation (interval (E) in the text).

The redox and P dynamics described above allow a timeline of environmental change, and its controls, to be reconstructed in the context of Snowball Earth deglaciation and the evolution of the GOE. We suggest, based on our data and others, an enhanced, rapid influx of P during Snowball Earth deglaciation, similar to that proposed for Neoproterozoic Snowball Earth events^57–60^. The consequent increase in productivity and organic carbon burial initially drove atmospheric and oceanic oxygenation during deposition of the middle Rooihoogte Formation (interval B; Fig. 1), but the oxygenation was transient (see below for further discussion). Nevertheless, this oxygenation promoted oxidative weathering on the continents and an enhanced influx of sulphate (indicated by low pyrite δ^34^S values), which led to the development of water column euxinia during deposition of interval C (Fig. 3A), and consequently, intense P recycling back to the water column (as indicated by high C_org_/P_org_ and C_org_/P_reac_ ratios; Fig. 1).

The development of water column euxinia ultimately resulted in sulphate consumption via microbial sulphate reduction, resulting in lower seawater sulphate concentrations, as indicated by the progressive shift to higher δ^34^S values and a transition back to ferruginous water-column conditions in the lower part of interval D (Fig. 1). However, P recycling initially persisted (Figs. 1, 2) due to sufficient sulphate availability to drive pore-waters sulphidic, as indicated by moderate Fe_py_/Fe_HR_ ratios (Fig. S3). Ultimately, the positive productivity feedback imposed by P recycling across this interval further enhanced organic carbon production and burial (indicated by elevated C_org_ contents; Fig. 1), leading to a return to an oxygenated atmosphere across the Rooihoogte-Timeball Hill Formation boundary^8,10^. Furthermore, coupled to a probable progressive decrease in the dissolved P influx from continental weathering in the aftermath of glacial melting, the return of ferruginous seawater conditions ultimately resulted in the re-establishment of the P_Fe_ trap (Fig. 3B), as indicated by the subsequent transition back to low C_org_/P_reac_ ratios (Fig. 1). This negative nutrient feedback imposed by P drawdown in association with Fe minerals resulted in decreased water-column oxygen demand, through lower organic carbon production, and hence better oxygenated shallow waters.

Following these post-glacial dynamics, under oxic, dysoxic and ferruginous conditions through the remainder of intervals D and E, P recycling was limited or absent, despite occasionally elevated C_org_/P_org_ and P/Al ratios (Fig. 1). These elevated C_org_/P_org_ and P/Al ratios likely reflect enhanced P drawdown in association with Fe minerals under transient ferruginous conditions (as indicated by relatively high P_Fe_ contents for these samples; Table S4). In all cases, however, low C_org_/P_reac_ ratios support efficient sequestration of P in the sediment, either via the formation of authigenic minerals such as CFA^33^ or via trapping of upward-migrating P by iron minerals^61^. This sequestration of P would have restricted oxygen production, leading to atmospheric oxygen concentrations that were likely persistently low (potentially straddling the 10^−6^ PAL threshold). However, within this framework, a balance between low rates of productivity-driven oxygen production and consequent oxidation of atmospheric methane may have driven a progressive descent into the final, regional Paleoproterozoic glaciation^1,42^. This sequence of events is remarkably similar to those that occurred in the aftermath of the Neoproterozoic Sturtian glaciation^52^. Following the initial Sturtian deglaciation, a shift to euxinic conditions was followed by a transition to oxygenated conditions. For the majority of the studied locations, there is also a pronounced shift to highly elevated C:P ratios for both organic and reactive P, followed by a return to lower values^52^.

### Constraining oxygen fluctuations during the GOE

**Fig. 4:**
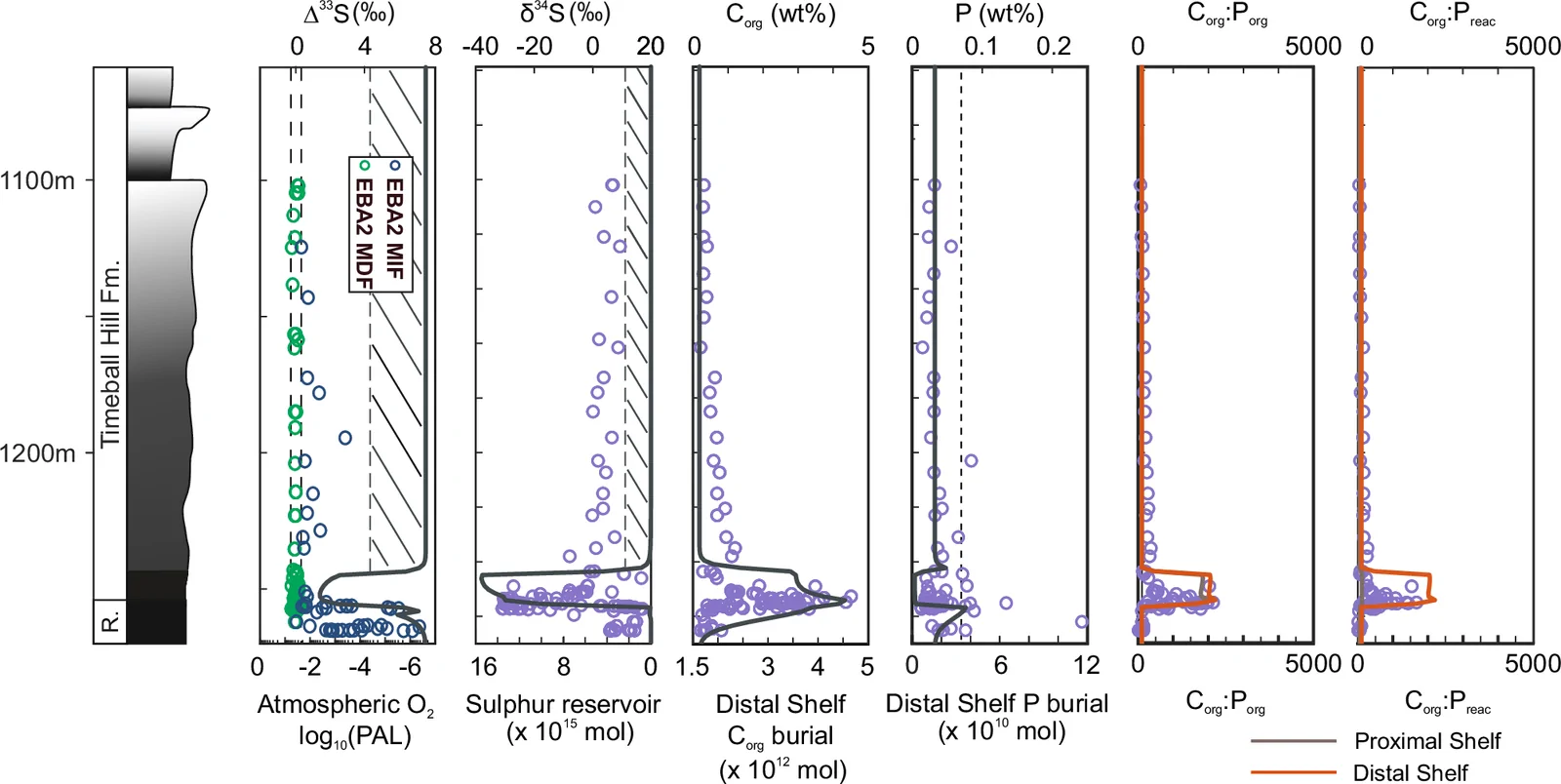
Biogeochemical modelling results. The Δ^33^S and δ^34^S records are compared to the modelled atmospheric oxygen and sulphur reservoir sizes. Note that the modelled atmospheric O_2_ and sulphur reservoir scales are reversed to aid comparison with the geochemical data. Organic carbon (C_org_) and total phosphorus (P) contents are compared to the organic carbon and phosphorus burial rates on the simulated continental shelf. C_org_:P_org_ and C_org_:P_reac_ ratios are compared directly to modelled C:P ratios, with both the proximal and distal-shelf environment values shown. C_org_ – organic carbon, P_org_ – organic bound phosphorus, P_reac_ - reactive phosphorus. R.- Rooihoogte Formation.

To further evaluate the proposed feedbacks described above, we utilise an adapted biogeochemical model framework^62^ for the C, O, P and S cycles (Fig. 4; see Methods). The model was setup for generalised pre-GOE conditions through setting riverine phosphate inputs, and mantle and metamorphic reductant inputs, within established bounds to maintain a functionally anoxic atmosphere^25^. Phosphorus recycling rates in the model are assumed to be determined by a combination of both the pyrite burial rate and bottom-water oxygen content, reflecting the overall availability of sulphide in the water-column and pore-waters (see Methods). A post-glacial P and S weathering influx was approximated by back-calculating the original (prior to any recycling) P content of the sediment using the Redfield ratio and organic C contents, with the newly approximated P/Al ratios being compared to the average shale value^63^ (see Methods).

The model results indicate that the proposed post-glacial pulse of P and sulphate would have led to a substantial increase in productivity and atmospheric oxygen concentration during deposition of the Rooihoogte Formation (Fig. 4). However, as discussed above, an initial interval of atmospheric oxygenation was apparently interrupted by a transient return to anoxia in the upper Rooihoogte Formation (Fig. 1). This may be explained by a delayed pulse of degassing associated with the depressurisation of magmatic chambers following deglaciation^64,65^. While there is uncertainty in this depressurisation mechanism even for the Pleistocene glaciations, particularly with regard to the overall magnitude and timing of degassing^66^, our model results suggest that a delayed pulse of reduced gases would have been able to briefly return the atmosphere to anoxia, and when combined with the post-glacial weathering input, produces model outputs that are in close agreement with the geochemical data (Fig. 4). Specifically, the modelled atmospheric O_2_ and S reservoir dynamics match the sulphur isotope (Δ^33^S and δ^34^S) records reasonably well, while the distal shelf C_org_, P and C:P ratios also respond in a similar manner to the data, with a rapid rise in C_org_ and C:P ratios associated with increased pyrite burial during the second oxygenation interval.

Importantly, having established the efficacy of the model, we are able to place constraints on the magnitude of O_2_ fluctuations and their controls (Fig. 4). Here, the initial rise in atmospheric oxygen in the middle of the Rooihoogte Formation, prior to delayed magmatic degassing, was driven by the increased P input, productivity and carbon burial following glacial melting, and this may have led to a relatively minor increase in atmospheric O_2_ concentration ( > 10^−6^ PAL). By contrast, the additional contribution to productivity and carbon burial from P recycling during the subsequent canonical loss of MIF-S at the height of post-glacial weathering (i.e., across the Rooihoogte-Timeball Hill Formation boundary) likely led to a major rise in atmospheric O_2_, with concentrations potentially approaching 10^−2^ PAL.

While we do not directly consider more complex atmospheric oxygen and methane dynamics during deposition of the overlying section, our reconstruction of P cycle dynamics highlights that a major drop in atmospheric O_2_ concentration would be expected^1^, due to the negative productivity feedback initiated by P sequestration into the sediments with the development of ferruginous conditions (Fig. 3B). Indeed, the full suite of data presented here suggests that minor changes in the balance between the fluxes of reductants and oxygen production would have been capable of readily driving variability in atmospheric oxygen levels at concentrations that may have straddled the 10^−6^ PAL MIF-S to MDF-S threshold. This possibility is supported by atmospheric modelling results, which have demonstrated that stable oxic and anoxic atmospheric states were both achievable, with rapid transitions between these states being dependent on the redox composition and fluxes of gases with respect to reservoir sizes^67,68^.

Our approach documents the impact of deglaciation on the behaviour of the P cycle within the context of the oscillatory rise of oxygen across the GOE. Glacial meltwaters brought appreciable bioavailable phosphate from the continents, ultimately driving an initial moderate increase in atmospheric O_2_ concentration. This led to enhanced oxidative weathering of the continents and hence an increased influx of sulphate, leading to the development of localised water-column euxinia and extensive P recycling. These feedbacks led to a major rise in atmospheric O_2_, however, as the rate of pyrite burial exceeded the rate of sulphate supply from oxidative weathering, the ocean transitioned back to a ferruginous state. This ultimately resulted in P drawdown, which, alongside a waning post-glacial influx of P, led to decreased net oxygen production and a newly established quasi-stable state. Subsequently, prior to the final, regional glaciation^42^, oxygen concentrations were maintained at an overall low level, but with oscillations potentially being driven by a changing balance between reductant inputs and oxygen production. The consistent, dynamic biogeochemical behaviour we observe between the phosphorus, carbon, sulphur and oxygen cycles suggests that the extreme climatic fluctuations that were a defining characteristic of the GOE drove dramatic variability in atmospheric and seawater oxygenation levels, with this instability persisting for over 200 million years.

## Methods

### Geochemical analyses

Samples were collected from the well-preserved EBA-2 drill-core at the National Core Library in Donkerhoek, South Africa (see Supplementary Information for a full description of the geological setting). Sulphur isotope and C_org_ concentration data were previously published^1^. Redox-sensitive trace elements (U, Mo, Re) and major elements (P and Al) were determined on ashed samples (450 °C overnight), followed by a HNO_3_–HF–HClO_4_–H_3_BO_3_ digest (full details, including precision and accuracy, are reported in the Supplementary Information).

Phosphorus phase partitioning was conducted following a revised extraction procedure for ancient rocks^69^. Four operationally-defined P phases were targeted, including iron-bound P (P_Fe_), authigenic P (P_aut_), organic-bound P (P_org_) and crystalline apatite (predominantly detrital P; P_cryst_). Phosphorus concentrations were either determined spectrophotometrically using the molybdate-blue method at 880 nm^70^, or via ICP-OES for extracts containing chemical compounds that interfere with colour development via the molybdate-blue method^69^. Reactive P (P_reac_) is quantified as the sum of P_Fe_, P_aut_ and P_org_^31^. Full details, including precision and accuracy, and consideration of the potential impact of transfer of P_reac_ to P_cryst_, are reported in the Supplementary Information.

### Biogeochemical Modelling

We utilize a 5-box ocean-atmosphere biogeochemical model for the C-O-P cycles (modified from ref. ^62^), supplemented by a 1-box marine sulphur cycle, to examine the response of the phosphorus cycle to a changing seawater sulphate reservoir and sulphur fluxes. This S cycle framework is based upon the single-box COPSE model framework^71^. Both frameworks are briefly summarised here, with a breakdown of the model fluxes and reservoir calculations provided in Tables S1–3. The model consists of four ocean boxes^62^, representing the proximal and distal shelf along with the surface and deep oceans, and within them, the organic C, organic P, soluble P and oxygen cycles are tracked. An atmosphere box is also included, which only considers oxygen. The P cycle includes the three major burial phases of P, namely organic-bound P (P_org_), iron-oxide-bound P (P_Fe_) and authigenic P (P_aut_).

The addition of the S cycle includes a single box encompassing all regions of the ocean, where reduced and oxidized forms of S are included. Inputs of S include pyrite weathering, which is calculated based on a previously published oxidative weathering relationship (^72^; Eqn. S76). This assumption allows for all pyrite to be oxidized when pO_2_ is greater than 0.35% PAL (transport-limited regime), with almost all pyrite remaining unoxidized at pO_2_ levels lower than ~3 × 10^−4^% PAL (reaction-limited)^72^. We also consider the assumption^73^ that only 60% of continental pyrite could be weathered due to the absence of land plants. Reduced sulphur degassing is also considered in a similar formulation to COPSE^71^, but without the assumption of a changing crustal pyrite inventory, given the relatively short run-time of this model (Eqn. S78). Pyrite weathering is considered a sink of oxygen in the same manner as the oxidative weathering of organic matter. Gypsum weathering and oxidised sulphur degassing are not considered here, as the earliest reported appreciable sulphate evaporite deposits are at ~2.4 Ga^16,17,74^, meaning that if gypsum was extensively buried at this time, it would likely not have been recycled through subduction and uplift in time to be weathered and metamorphically degassed during deposition of the studied interval. Burial of reduced S as pyrite is calculated based on both organic C availability (organic C burial flux) and the S reservoir size, following COPSE (Eqn. S80). This pyrite burial flux is then introduced as an oxygen source to the atmosphere^71^. A reduced gas input appropriate for pre-GOE conditions is imposed on the model, as in previous work^25,62^.

A pulse of both P and sulphate through post-glacial weathering is introduced under the assumption that this immediately followed the Snowball Earth glaciation. This input flux is calculated based on the initial burial proportion of organic C and P in order to back-calculate a P/Al ratio of the sediments prior to diagenetic recycling. These ratios are then compared to the Phanerozoic average shale P/Al ratio in order to provide average and maximum changes relative to the present day. By distinguishing between oxic and euxinic samples, minimal and maximum input fluxes can be estimated. This amounts to 70% of the present-day P input prior to and following deglaciation, and values ranging from 196 to 733% of the present-day input represent the post-glacial pulse. Based on this range, and informed by our dataset, we use a conservative input of 170%. This flux was then applied over a period within estimates of Snowball Earth deglaciation rates ( ~ 100 kyrs^75^). In addition to this, we include the impact of depressurized magma chambers during large-scale deglaciation, leading to an excess pulse of magmatic gases^64^. This was included as an additional input of reduced gases to the atmosphere (10 × 10^12^) and aids in reproducing the initial rapid loss and subsequent return of the MIF-S signal during deposition of the Rooihoogte Formation.

## Supplementary information

Supplementary Information

Transparent Peer Review file

## Data Availability

The data generated in this study are provided in the Supplementary Information and is available in the Figshare repository https://doi.org/10.6084/m9.figshare.32895593^76^.

## Code availability

Code is available at https://github.com/lalcott/oneboxScycle.git and https://doi.org/10.5281/zenodo.21162169^77^.
