# Supplementary material for: A nutrient control on oxygenation dynamics during Earth’s Great Oxidation Episode
Source: Nat Commun. 2026 Aug 27;17:10294. doi: 10.1038/s41467-026-76597-y (PMC13620156; doi:10.1038/s41467-026-76597-y)
Supplement: Supplementary file 1 [file 41467_2026_76597_MOESM2_ESM.pdf]

Peer Review File

A nutrient control on oxygenation dynamics during Earth's Great Oxidation Episode

Corresponding Author: Dr Lewis Alcott

This file contains all reviewer reports in order by version, followed by all author rebuttals in order by version.

Version 0:

Reviewer comments:

Reviewer #1

(Remarks to the Author)
This study presents high-quality data, in-depth interpretation, and robust model to investigate the dynamic feedback mechanisms between the P cycle and atmospheric/oceanic redox fluctuations during the GOE. By combining high-resolution geochemical data with biogeochemical modeling, the researcher systematically reconstructs the coupled evolution of O-P-S-C cycles recorded in the Late Paleoproterozoic (~2.32–2.25 Ga) Transvaal Supergroup in South Africa. This paper is interesting, important, and organized well, and thus I suggest publication after minor revision.

Major Comments
1. Impacts of early diagenesis and late metamorphic alteration on P speciation data should fully considered
2. Some samples from the upper Timeball Hill Formation record higher P/Al and Corg/Porg ratios, but the Corg/Preact ratios are close to the Redfield ratio. This should be discussed but is ignored.

Minor Comments
Line 34. I think it should be "glaciation" rather than "deglaciation".
Line 41–42. "transition from … to a permanently oxygenated atmosphere".
Line 41–42. It's better to clarify the evolution of seawater redox chemistry and biological evolution.
Line 51. "Global stratigraphic correlations"?
Line 67–69: Rephrasing the sentence structure to avoid ambiguity.
Line 86. Introduce what samples are.
Line 87–88. You mean the final global glaciation and the later regional glaciation?
Line 116–117. Here you'd better provide your and previous reconstruction results, and then you can state they are consistent.
Line 120. "Briefly" rather than "Specifically".
Line 124. A brief summary sentence would help readers better understand how to use these ratios (Re/Mo, Re/U, and Mo/U) to reconstruct paleo-redox condition.
Line 127–128. I suggest to provide Fe speciation profile in Figure 1.
Line 135–136. How about your data (Re/Mo, Re/U, and Mo/U)?
Line 151–153. The Rooihoogte-Timeball Hill boundary is lower (was deposited earlier) than the upper Timeball Hill Formation.
Line 153. Add "P" between "extensive" and "drawdown".
Line 170. "S isotope composition" or "δ34S value".
Line 192. I am sorry that I don't understand how you get this conclusion (of course I agree with it). I just can see that P was back released into water column during the deposition of the Rooihoogte-Timeball Hill boundary based on your own data.
Line 193–194. Increases in productivity and organic carbon burial are not supported by your data here.
Line 200–201. Just consumption of seawater sulfate.
Line 202–203. Fepy/FeHR ratios are still high, although they reflect ferruginous water-column. This also suggests the consumption of seawater sulfate.
Line 218. Corg/Preact ratios are still low, which are not explained. See Major Comment 1.

(Remarks on code availability)

Reviewer #2

(Remarks to the Author)
Comments on the manuscript entitled ‘A nutrient control on oxygenation dynamics during Earth’s Great Oxidation Episode’, by Alcott et al.

In this contribution, the authors examine the links and feedbacks between ocean redox chemistry, phosphorus recycling and atmospheric oxygen production at the aftermath of the final GOE glaciation. They apply newly developed redox proxies and a P phase partitioning approach adapted to ancient sedimentary rocks to this tremendously important period in Earth’s history. In addition, they use geochemical modelling to support their geochemical dataset. The study is highly topical and will be welcome by the broad community.

I find the manuscript very well written and the story really convincing. The authors did a great job explaining a complex dataset. I only have a few suggestions that I hope will improve the clarity in some cases. I have a minor disagreement with the differential removal mechanisms for Mo and U under ferruginous conditions, but this is part of the scientific debate and I have no major problem with the current interpretation made by the authors. I recommend for publication in Nature Communications.

Detailed comments:

Fig. 1: The stratigraphic log is poorly explained. What do the dots mean? Sandstone?

L116: ‘based on the dominant atmospheric and oceanic redox state’. Unclear: from the figure it seems that the ocean redox was considered to define these stages (A-E). How was the atmospheric redox state used here?

L123: ‘a 123 particulate shuttle mechanism whereby Mo (but not U or Re) is sequestered due to uptake by Fe minerals’. In my understanding, this is highly dependent on the Fe mineralogy. Adsorption onto ferric particles seem to favour Mo drawdown over U. However, U can be readily reduced by ferrous or mixed-valence particles such as green rust (e.g., O’Loughlin et al., 2003), with subsequent drawdown as uraninite… As for now, the author’s interpretation is the most popular one, but I think it is worth considering that the nature of ocean Fe particles in the Archean or the Proterozoic might modify that differential behaviour between Mo and U under ferruginous conditions.

L130 and Figs. S2 and S3: the authors suggest that stage A is characterized by fluctuations between oxic and ferruginous conditions, but Fig. S3 also shows elevated Fe_{py}/Fe_{HR}. Do these specific samples plot within the oxic domain (Fe_{HR}/Fe_T <0.22)? Perhaps you could rephrase as ‘However, occasionally elevated Fe_{HR}^*/Fe_T and Fe_T/Al ratios (but low Fe_{py}/Fe_{HR}; figs. S2 and S3)’.

L147-148: I struggle to find evidence for ‘more persistently ferruginous conditions leading up to, and during the final regional glaciation’. There seems to be only one data point with Fe_{HR}^*/Fe_T >0.38, and with the scale of the Fe/Al plot, it is hard to see whether there are any Fe/Al enrichments at all (I also suggest to change the scale of that plot). Also, out of interest, did you examine the diamictite’s matrix?

L102: ‘formation’ or ‘Formation’? It seems inconsistent throughout.

L164-169: this very long sentence is also very hard to follow (the word ‘associated’ is repeated 3 times…), please rephrase.

L165 and L167: ‘very low values’… ‘elevated sulfate’: can you please specify the range in both cases?

Fig. 2: It is unclear to me what samples are considered for this figure. I understand this is across the Rooihoogte-Timeball Hill formation boundary but it would be worth explaining clearly what depths were considered.

Fig. 3: I wonder If it is possible to indicate in the figure caption the stages defined in Fig. 1 (A-B-C-D or E) that correspond to each scenario? That would enhance clarity.

(Remarks on code availability)

Reviewer #3

(Remarks to the Author)
Review of the manuscript by Alcott et al. submitted to Nature Communications: “A nutrient control on oxygenation dynamics during Earth’s Great Oxidation Episode”
In this manuscript, the authors present phosphorus (P) phase partitioning and redox-sensitive trace-element data from the geologic section that records the oxygenation history across the Great Oxidation Event. They present evidence for a link between deglaciation and oxygen evolution during this period, which is linked to the recycling of P from sediments associated with the variations in the global sulfur cycle. The manuscript is well written, and I have one major comment and some minor ones.

Major Comments:
L163–172: “As a first-order assessment of...”
The reviewer thinks that further justification would be needed to discuss the relationship between sulfate concentration and S-isotope signatures, even if this is a first-order assessment. As the authors briefly mentioned in the manuscript, the pyrite δ34S record would be affected by processes other than variations in the global sulfur cycle (e.g., depositional environments, local temperature, etc.) (e.g., Pasquier et al., 2017; 2021; Halevy et al., 2023). While the negative shift in δ34S may be related to changes in the oceanic sulfate reservoir, this large shift may also be caused by environmental changes, as in the case of the Pleistocene pyrite δ34S (e.g., Pasquier et al., 2017). Under the Archean-Proterozoic condition, the exit pathway of marine S would have predominantly been pyrite burial (Fike et al., 2015; Fakhraee et al., 2024). This means that bulk pyrite deposited from the ocean would be close to the mantle-derived volcanic δ34S, which is near 0‰ (Fike et al., 2015), indicating that at a steady state, bulk pyrite δ34S would have been near 0‰ regardless of marine sulfate concentration. The bulk pyrite δ34S value can be negative in the aftermath of a glaciation, but sulfate concentration maximum can lag the pyrite δ34S minimum (e.g., Figure 3 of Harada et al., 2025). In addition, it is uncertain to the reviewer how the very high positive δ34S signal in the A–C interval was produced.

Minor Comments:
L66–67: “final global glaciation”
The reviewer thinks that the Paleoproterozoic global glaciation event (i.e., snowball Earth) is confirmed only in the Makganyene Formation (Evans et al., 1997), which is associated with the first glaciation in the Huronian Supergroup. The other glaciations may have been global, but this has not been confirmed, so the “final” global glaciation in the Paleoproterozoic sounds strange to the reviewer.

L58–62: “Going further, the…”
The relationship between global glaciation, the nutrient cycle, and atmospheric oxygen has also been investigated using theoretical models (Harada et al., 2015; 2025; Ruiz et al., 2023; 2024).

L200: “The development of water column euxinia...”
A more detailed explanation of why the development of water-column euxinia results in sulfate drawdown would be necessary.

L214: “Following these post-glacial…”
How does it resemble the aftermath of the Sturtian glaciation?

L241–243: “This may be explained...”
It is very interesting to apply the mechanism proposed by Huybers and Langmuir (2009) for Pleistocene glacial cycles to this context. However, the magnitude of this mechanism is difficult to test even in the Pleistocene because the mechanisms of atmospheric CO2 variations during glacial cycles have not yet been fully understood, although investigations using theoretical models (e.g., Ganopolski and Brovkin, 2017; Köhler, 2023; Kobayashi et al., 2021) are advancing understanding. The reviewer agrees that the pressure change during deglaciation could result in a decline in atmospheric oxygen, but the magnitude and timing of this mechanism are highly uncertain, and this uncertainty could be discussed more carefully by noting it in the manuscript.

L246: “Specifically, the modelled…”
Same as my comment to L163–172.

Figure 1:
It would be helpful if the glacial period is shown in the figure.

Figure 2:
Same as my comment to L163–172, but it is questionable that “increased sulfate availability” is directly related to pyrite δ34S.

References
Evans, D. A., Beukes, N. J., & Kirschvink, J. L. (1997). Low-latitude glaciation in the Palaeoproterozoic era. Nature, 386(6622), 262-266.
Fakhraee, M., Crockford, P. W., Bauer, K. W., Pasquier, V., Sugiyama, I., Katsev, S., ... & Planavsky, N. (2025). The history of Earth’s sulfur cycle. Nature Reviews Earth & Environment, 6(2), 106-125.
Fike, D. A., Bradley, A. S., & Rose, C. V. (2015). Rethinking the ancient sulfur cycle. Annual Review of Earth and Planetary Sciences, 43, 593-622.
Ganopolski, A., & Brovkin, V. (2017). Simulation of climate, ice sheets and CO2 evolution during the last four glacial cycles with an Earth system model of intermediate complexity. Climate of the Past, 13(12), 1695-1716.
Halevy, I., Fike, D. A., Pasquier, V., Bryant, R. N., Wenk, C. B., Turchyn, A. V., ... & Claypool, G. E. (2023). Sedimentary parameters control the sulfur isotope composition of marine pyrite. Science, 382(6673), 946-951.
Harada, M., Tajika, E., & Sekine, Y. (2015). Transition to an oxygen-rich atmosphere with an extensive overshoot triggered by the Paleoproterozoic snowball Earth. Earth and Planetary Science Letters, 419, 178-186.
Harada, M., Miura, Y., Watanabe, Y., & Ozaki, K. (2025). Redox Dynamics of the Atmosphere and Oceans Induced by the Paleoproterozoic Snowball Earth Events. Geobiology, 23(6), e70040.
Huybers, P., & Langmuir, C. (2009). Feedback between deglaciation, volcanism, and atmospheric CO2. Earth and Planetary

Science Letters, 286(3-4), 479-491.
Kobayashi, H., Oka, A., Yamamoto, A., & Abe-Ouchi, A. (2021). Glacial carbon cycle changes by Southern Ocean processes with sedimentary amplification. Science Advances, 7(35), eabg7723.
Köhler, P. (2023). Atmospheric CO2 concentration based on boron isotopes versus simulations of the global carbon cycle during the Plio-Pleistocene. Paleoceanography and Paleoclimatology, 38(2), e2022PA004439.
Pasquier, V., Sansjofre, P., Rabineau, M., Revillon, S., Houghton, J., & Fike, D. A. (2017). Pyrite sulfur isotopes reveal glacial– interglacial environmental changes. Proceedings of the National Academy of Sciences, 114(23), 5941-5945.
Pasquier, V., Bryant, R. N., Fike, D. A., & Halevy, I. (2021). Strong local, not global, controls on marine pyrite sulfur isotopes. Science advances, 7(9), eabb7403.
Ruiz, D. G., Goldblatt, C., & Ahm, A. S. (2023). Climate shapes the oxygenation of Earth's atmosphere across the Great Oxidation Event. Earth and Planetary Science Letters, 607, 118071.
Ruiz, D. G., Goldblatt, C., & Ahm, A. S. (2024). Climate variability leads to multiple oxygenation episodes across the Great Oxidation Event. Geophysical Research Letters, 51(3), e2023GL106694.

(Remarks on code availability)
I note that the link for the code provided by the authors did not work.

Open Access This Peer Review File is licensed under a Creative Commons Attribution 4.0 International License, which permits use, sharing, adaptation, distribution and reproduction in any medium or format, as long as you give appropriate credit to the original author(s) and the source, provide a link to the Creative Commons license, and indicate if changes were made.
In cases where reviewers are anonymous, credit should be given to 'Anonymous Referee' and the source.
The images or other third party material in this Peer Review File are included in the article’s Creative Commons license, unless indicated otherwise in a credit line to the material. If material is not included in the article’s Creative Commons license and your intended use is not permitted by statutory regulation or exceeds the permitted use, you will need to obtain permission directly from the copyright holder.
To view a copy of this license, visit https://creativecommons.org/licenses/by/4.0/

REVIEWER COMMENTS

Reviewer #1 (Remarks to the Author):

This study presents high-quality data, in-depth interpretation, and robust model to investigate the dynamic feedback mechanisms between the P cycle and atmospheric/oceanic redox fluctuations during the GOE. By combining high-resolution geochemical data with biogeochemical modeling, the researcher systematically reconstructs the coupled evolution of O-P-S-C cycles recorded in the Late Paleoproterozoic (~2.32–2.25 Ga) Transvaal Supergroup in South Africa. This paper is interesting, important, and organized well, and thus I suggest publication after minor revision.

We would like to thank the reviewer for their positive comments.

Major Comments

1. Impacts of early diagenesis and late metamorphic alteration on P speciation data should fully considered

We initially addressed this issue in the Supplementary Information. We have further expanded our discussion on sink switching of P phases during diagenesis and metamorphism in the SI:

"It is important to acknowledge the potential role diagenesis and metamorphic alteration can play in the interpretation of P phase partitioning data. Iron bound-P and organic P can be readily released to solution during early diagenesis[32,33,62]. However, any P that remains in the sediment, either through transfer to carbonate fluorapatite or vivianite, or re-adsorption to Fe-(oxyhydr)oxides, is accounted for as part of the overall reactive P pool (P_{reac}). Of greater potential impact is the possible phase transfer of P_{aut} to P_{cryst}, which has specifically been identified as a factor to consider when utilizing P phase partitioning data for ancient sediments[70,98], whereby the conversion of P_{aut} to P_{cryst} would lead to an increase in C_{org}:P_{reac} ratios. However, even in the highly unlikely scenario that all P_{cryst} was originally present as P_{aut}, and thus P_{reac} equates to total P (P_{tot}), C_{org}:P_{tot} ratios at the Rooihoogte-Timeball Hill boundary still plot above 106:1 (Fig. S4), unequivocally confirming that recycling was occurring across this interval. Therefore, our conclusions are not impacted by possible limited transfer of P_{aut} to P_{cryst}."

2. Some samples from the upper Timeball Hill Formation record higher P/Al and Corg/Porg ratios, but the Corg/Preact ratios are close to the Redfield ratio. This should be discussed but is ignored.

Very few samples have higher values for both P/Al and C_{org}/P_{org}. Occasionally elevated P/Al values correspond to samples with relatively elevated P_{Fe} and P_{aut}, suggesting additional

drawdown in association with Fe minerals under transient ferruginous conditions. We now discuss this on Lines 225-231:

"Following these post-glacial dynamics, under oxic, dysoxic and ferruginous conditions through the remainder of intervals D and E, P recycling was limited or absent, despite occasionally elevated C_{org}/P_{org} and P/Al ratios (Fig. 1). These elevated C_{org}/P_{org} and P/Al ratios likely reflect enhanced P drawdown in association with Fe minerals under transient ferruginous conditions (as indicated by relatively high P_{Fe} contents for these samples; Table S4). In all cases, however, low C_{org}/P_{reac} ratios support efficient sequestration of P in the sediment, either via the formation of authigenic minerals such as CFA[33] or via trapping of upward-migrating P by iron minerals[62]."

Minor Comments

Line 34. I think it should be "glaciation" rather than "deglaciation".

Yes, this is correct. Changed as suggested.

Line 41–42. "transition from … to a permanently oxygenated atmosphere".

This has been edited as suggested.

Line 41–42. It's better to clarify the evolution of seawater redox chemistry and biological evolution.

The detailed evolution of seawater redox chemistry after the GOE is very complex, so we prefer to keep this simple here, but we have added text about the role of oxygen in biological evolution:

"The Great Oxidation Episode (GOE) documents the transition to a permanently oxygenated atmosphere[1], and represents a defining interval in Earth history with respect to the subsequent evolution of seawater redox chemistry and the role of marine oxygenation in the course of biological evolution[2-4]."

Line 51. "Global stratigraphic correlations"?

This has been edited as suggested.

Line 67–69: Rephrasing the sentence structure to avoid ambiguity.

This has been rephrased to: "*These redox dynamics would have greatly impacted the bioavailability of phosphorus (P), the ultimate limiting nutrient on geological timescales[24], which is thought to have been fundamental in priming and driving the GOE[25,26]."*

Line 86. Introduce what samples are.

We have now noted that the samples considered were shale.

Line 87–88. You mean the final global glaciation and the later regional glaciation?

Yes, this is correct and has been altered accordingly.

Line 116–117. Here you'd better provide your and previous reconstruction results, and then you can state they are consistent.

We go on to discuss our and others redox interpretations following the discussion on how we interpret the RSTEs on lines 112-128.

Line 120. "Briefly" rather than "Specifically".

This has now been edited as suggested.

Line 124. A brief summary sentence would help readers better understand how to use these ratios (Re/Mo, Re/U, and Mo/U) to reconstruct paleo-redox condition.

We have now added some additional text to further discuss the interpretation of these ratios on lines 118-128.

"In brief, U sequestration commonly occurs under anoxic bottom-water conditions[43], while Re is more sensitive to oxygen depletion and begins to be sequestered under dysoxic conditions[44,45]. By contrast, substantial drawdown of Mo either requires anoxia and the presence of dissolved sulphide, or a particulate shuttle mechanism whereby Mo (but not U or Re) is sequestered due to uptake by Fe minerals precipitating at the redoxcline above deeper ferruginous waters[46-48]. Therefore, high Re/U and Re/Mo values commonly indicate dysoxic conditions (further supported by high Re/Al and low highly reactive (Fe_{HR}) to total Fe (Fe_T) values), whereas low Re/Mo and high Re/U values commonly develop under anoxic, ferruginous conditions (supported by high Re/Al, U/Al and Fe_{HR}/Fe_T and low pyrite (Fe_{py}) to Fe_{HR} values)[37]. Euxinic conditions are commonly characterized by high Mo/U values, alongside high Fe_{HR}/Fe_T and Fe_{py}/Fe_{HR} values (also supported by high Re/Al, U/Al and Mo/Al, and low Re/Mo and Re/U values)[37]."

Line 127–128. I suggest to provide Fe speciation profile in Figure 1.

In an effort to keep within the page sizing, we summarize the findings from the iron speciation profile within the Ocean Redox panel on Figure 1. The iron speciation data are not new to this manuscript and have been published previously (Poulton et al., 2021), and we prefer to keep these data in the SI. Our new trace metal data are consistent with the Fe speciation data, but the new aspect is the identification of dysoxic conditions.

Line 135–136. How about your data (Re/Mo, Re/U, and Mo/U)?

As noted on line 141, there is limited available redox data. We do however already include the interpretation of Mo/U in interval B, which is overall supported by redox data from a coeval drill core (Poulton et al., 2021).

Line 151–153. The Rooihoogte-Timeball Hill boundary is lower (was deposited earlier) than the upper Timeball Hill Formation.

This has been rearranged for clarity as suggested.

Line 153. Add “P” between “extensive” and “drawdown”.

This has been added as suggested.

Line 170. “S isotope composition” or “δ34S value”.

This has been edited as *sulphur isotope composition* as suggested.

Line 192. I am sorry that I don’t understand how you get this conclusion (of course I agree with it). I just can see that P was back released into water column during the deposition of the Rooihoogte-Timeball Hill boundary based on your own data.

We have now included the note that this interpretation includes not only our own data, but also other investigations of Snowball Earth deglaciations as well. Whilst the redox recycling of P is supported by our data, data from others proposes and supports an enhanced influx of P during Snowball Earth deglaciation.

Line 193–194. Increases in productivity and organic carbon burial are not supported by your data here.

We note that the rise in organic carbon burial occurs in interval B, which can be seen in the data. The modelling demonstrates that a progressive ‘ramping up’ in organic carbon burial also fits our interpretation.

Line 200–201. Just consumption of seawater sulfate.

We have edited the sentence as suggested.

Line 202–203. Fepy/FeHR ratios are still high, although they reflect ferruginous water-column. This also suggests the consumption of seawater sulfate.

We note this in the following line in which sulfidic porewaters persist.

Line 218. Corg/Preact ratios are still low, which are not explained. See Major Comment 1.

We have now dealt with the question in the response to the second major response above.

Reviewer #2 (Remarks to the Author):

Comments on the manuscript entitled 'A nutrient control on oxygenation dynamics during Earth's Great Oxidation Episode', by Alcott et al.

In this contribution, the authors examine the links and feedbacks between ocean redox chemistry, phosphorus recycling and atmospheric oxygen production at the aftermath of the final GOE glaciation. They apply newly developed redox proxies and a P phase partitioning approach adapted to ancient sedimentary rocks to this tremendously important period in Earth's history. In addition, they use geochemical modelling to support their geochemical dataset. The study is highly topical and will be welcome by the broad community.

I find the manuscript very well written and the story really convincing. The authors did a great job explaining a complex dataset. I only have a few suggestions that I hope will improve the clarity in some cases. I have a minor disagreement with the differential removal mechanisms for Mo and U under ferruginous conditions, but this is part of the scientific debate and I have no major problem with the current interpretation made by the authors. I recommend for publication in Nature Communications.

We are very grateful to the reviewer for the positive and supportive response.

Detailed comments:

Fig. 1: The stratigraphic log is poorly explained. What do the dots mean? Sandstone?

We have now better described the log illustrations in the legend and figure captions.

L116: 'based on the dominant atmospheric and oceanic redox state'. Unclear: from the figure it seems that the ocean redox was considered to define these stages (A-E). How was the atmospheric redox state used here?

It is actually a mixture of ocean and atmospheric redox state, based on the same intervals defined by Poulton et al. (2021). The sections A-C in the figure do not precisely correspond to changes in ocean redox alone. We thus prefer to keep this as it is.

L123: 'a 123 particulate shuttle mechanism whereby Mo (but not U or Re) is sequestered due to uptake by Fe minerals'. In my understanding, this is highly dependent on the Fe mineralogy. Adsorption onto ferric particles seem to favour Mo drawdown over U. However, U can be readily reduced by ferrous or mixed-valence particles such as green rust (e.g., O'Loughlin et al., 2003), with subsequent drawdown as uraninite… As for now, the author's interpretation is the most popular one, but I think it is worth considering that the nature of ocean Fe particles in the Archean or the Proterozoic might modify that differential behaviour between Mo and U under ferruginous conditions.

This is a very good point, and really requires knowledge of the primary depositional mineralogy. We have now included a consideration of the likely Fe particles and how they might impact Mo and U sequestration in the supplementary information (Lines 132-138).

"A variety of iron minerals may have driven the particulate shuttle during the Archean and Proterozoic. Iron- and Mn-(oxyhydr)oxides are often cited as the principal Mo shuttlers, but

within an anoxic ferruginous water column, other phases such as the mixed ferrous-ferric mineral, green rust[94], or ferrous minerals such as greenalite[95], may also have precipitated. However, iron silicates such as greenalite are a less efficient shuttle for Mo[96], suggesting that this phase was not responsible for elevated Mo contents in the sediments."

L130 and Figs. S2 and S3: the authors suggest that stage A is characterized by fluctuations between oxic and ferruginous conditions, but Fig. S3 also shows elevated Fepy/FeHR. Do these specific samples plot within the oxic domain (FeHR/FeT <0.22)? Perhaps you could rephrase as 'However, occasionally elevated FeHR*/FeT and FeT/Al ratios (but low Fepy/FeHR; figs. S2 and S3)'.

Thank you very much for highlighting this. We have edited the text with the reviewer's recommendation in mind.

L147-148: I struggle to find evidence for 'more persistently ferruginous conditions leading up to, and during the final regional glaciation'. There seems to be only one data point with FeHR*/FeT >0.38, and with the scale of the Fe/Al plot, it is hard to see whether there are any Fe/Al enrichments at all (I also suggest to change the scale of that plot). Also, out of interest, did you examine the diamictite's matrix?

While there are only two samples within interval E that have $Fe_{HR}*/Fe_T > 0.38$, there are several samples that lie within the unequivocal zone that require additional data to determine their redox state of deposition. With the supporting Fe/Al data, it is possible to determine that the samples were deposited in largely ferruginous conditions with some samples suggesting oxic conditions. We have now edited the Fe/Al plot in Figure S2, and included a logarithmic scale bar. We analysed very few diamictite samples, but where we did, it was the matrix that was specifically targeted.

L102: 'formation' or 'Formation'? It seems inconsistent throughout.

We have followed general procedure and use a capital when referring to a single formation, and lower case when referring to more than one formation.

L164-169: this very long sentence is also very hard to follow (the word 'associated' is repeated 3 times…), please rephrase.

We have rephrased the sentence as follows: *"Although there are a variety of potential controls on pyrite $\delta^{34}S$ values in ancient sediments[4,51,52], very low compositions (relative to the earlier Precambrian[4]) of about -35‰ across the Rooihoogte-Timeball Hill Formation boundary (Fig. 1) are consistent with elevated sulphate concentrations (above 200 µM[53])."*

L165 and L167: 'very low values'… 'elevated sulfate': can you please specify the range in both cases?

We have now quoted the lower value of $\delta^{34}S$ in our samples, as well as cross referenced this value to potential fractionations in Archean systems (Crowe et al., 2014; Habicht et al., 2002).

Fig. 2: It is unclear to me what samples are considered for this figure. I understand this is across the Rooihoogte-Timeball Hill formation boundary but it would be worth explaining clearly what depths were considered.

All available samples were considered in Figure 2. We have amended the figure caption to clarify.

Fig. 3: I wonder If it is possible to indicate in the figure caption the stages defined in Fig. 1 (A-B-C-D or E) that correspond to each scenario? That would enhance clarity.

We have now included the corresponding stages in the figure caption.

Reviewer #3 (Remarks to the Author):

Review of the manuscript by Alcott et al. submitted to Nature Communications: “A nutrient control on oxygenation dynamics during Earth’s Great Oxidation Episode”
In this manuscript, the authors present phosphorus (P) phase partitioning and redox-sensitive trace-element data from the geologic section that records the oxygenation history across the Great Oxidation Event. They present evidence for a link between deglaciation and oxygen evolution during this period, which is linked to the recycling of P from sediments associated with the variations in the global sulfur cycle. The manuscript is well written, and I have one major comment and some minor ones.

We are grateful for the supportive appraisal and helpful comments.

Major Comments:
L163–172: “As a first-order assessment of...”
The reviewer thinks that further justification would be needed to discuss the relationship between sulfate concentration and S-isotope signatures, even if this is a first-order assessment. As the authors briefly mentioned in the manuscript, the pyrite δ34S record would be affected by processes other than variations in the global sulfur cycle (e.g., depositional environments, local temperature, etc.) (e.g., Pasquier et al., 2017; 2021; Halevy et al., 2023). While the negative shift in δ34S may be related to changes in the oceanic sulfate reservoir, this large shift may also be caused by environmental changes, as in the case of the Pleistocene pyrite δ34S (e.g., Pasquier et al., 2017). Under the Archean-Proterozoic condition, the exit pathway of marine S would have predominantly been pyrite burial (Fike et al., 2015; Fakhraee et al., 2024). This means that bulk pyrite deposited from the ocean would be close to the mantle-derived volcanic δ34S, which is near 0‰ (Fike et al., 2015), indicating that at a steady state, bulk pyrite δ34S would have been near 0‰ regardless of marine sulfate concentration. The bulk pyrite δ34S value can be negative in the aftermath of a glaciation, but sulfate concentration maximum can lag the pyrite δ34S minimum (e.g., Figure 3 of Harada et al., 2025). In addition, it is uncertain to the reviewer how the very high positive δ34S signal in the A–C interval was produced.

We agree that the dominant exit pathway for marine S would have been as pyrite. However, once there was a reasonable pool of sulfate in the ocean (i.e., following the atmospheric oxygenation that these rocks document) only persistent, quantitative sulfate drawdown would result in a mantle pyrite $\delta^{34}S$ signature of ~0‰. This would require extensive, persistent euxinia, which is not evidenced by our redox data. Thus, under sulfate concentrations >200 μM (below which small fractionations commonly occur; Habicht et al., 2002), relatively large fractionations can occur. This has also been re-confirmed by Halevy et al. (2023), despite the earlier work of some of these authors that has commonly been reflected to be contrary to this, who note that increased seawater sulfate concentrations, as would be expected in association with the increased atmospheric oxygen concentrations at this time, drive increased isotopic fractionations in pyrite. These authors also note that more

subtle variations in fractionations may occur associated with other factors, which we entirely agree with. One prominent way in which this might occur is via a change in sedimentation rate (e.g., Pasquier et al., 2021). However, there is no sedimentological evidence for a change in sedimentation rate at this time, and instead, the precise links in the timing of pyrite $\delta^{34}S$ changes relative to rising atmospheric oxygen and ocean redox changes, strongly advocate these processes as being responsible for the trends observed. The somewhat heavy pyrite $\delta^{34}S$ signatures observed in Zone A likely reflect complete removal of porewater sulfate as pyrite under closed system conditions, which is consistent with our suggestion of enhanced diagenetic pyrite formation in this interval (new text on Lines 190-193 that notes relatively high Fe_{py}/Fe_{HR} ratios). The light pyrite $\delta^{34}S$ values in interval C correspond precisely with the major loss of the MIF-S signal, and hence most parsimoniously represent an increased sulfate influx from continental weathering, which would itself have driven the development of euxinia at this time, as observed in our data. As then documented by other Paleoproterozoic sediments during notable transitions to euxinic conditions (e.g., Shen et al., Am. J. Sci., 2003; Poulton et al., Nature, 2021), extensive drawdown of the still relatively low concentrations of sulfate in the ocean via enhanced pyrite burial under euxinic conditions, coupled with continued pyrite formation during closed system diagenesis, drove the return to heavy S isotope compositions in the lower part of interval D.

We thus maintain that our interpretation of these S isotope systematics matches the broader (and major) environmental perturbations occurring at this time. While the precise explanation for these pyrite S isotope values does not directly impact our conclusions related to P cycling, we nevertheless appreciate the reviewers comment that other potential pathways should not be dismissed out of hand, and have thus revised the text as follows:

"Although there are a variety of potential controls on pyrite $\delta^{34}S$ values in ancient sediments[4,51,52], very low compositions (relative to the earlier Precambrian[4]) of about -35‰ across the Rooihoogte-Timeball Hill Formation boundary (Fig. 1) are consistent with elevated sulphate concentrations (above 200 μM[53]). In considering alternative possibilities, we note that there is no sedimentological evidence for a change in sedimentation rate that might influence pyrite $\delta^{34}S$ values[54], and instead, the pyrite $\delta^{34}S$ trends occur coincident with major changes in atmospheric oxygenation and ocean redox conditions (Fig. 1). This suggests an enhanced flux of sulphate to the oceans (which increases $\delta^{34}S$ fractionation in pyrite[55,56]) driven by oxidative weathering of the continents linked to rising atmospheric oxygen[8]."

Minor Comments:
L66–67: "final global glaciation"
The reviewer thinks that the Paleoproterozoic global glaciation event (i.e., snowball Earth) is confirmed only in the Makganyene Formation (Evans et al., 1997), which is associated with the first glaciation in the Huronian Supergroup. The other glaciations may have been global, but this has not been confirmed, so the "final" global glaciation in the Paleoproterozoic sounds strange to the reviewer.

We have now refined our wording to allow for more interpretations with respect to the number of global glaciations, by simply referring to them as either 'widespread', as seems to be the case for the lower 3 glaciations, or 'regional' as is the case for the final glaciation.

L58–62: "Going further, the…"
The relationship between global glaciation, the nutrient cycle, and atmospheric oxygen has

also been investigated using theoretical models (Harada et al., 2015; 2025; Ruiz et al., 2023; 2024).

We have now included these references throughout the text.

L200: "The development of water column euxinia..."
A more detailed explanation of why the development of water-column euxinia results in sulfate drawdown would be necessary.

We have now amended the sentence to read *"The development of water column euxinia ultimately resulted in sulphate consumption via microbial sulphate reduction, resulting in lower seawater sulphate concentrations…"*

L214: "Following these post-glacial…"
How does it resemble the aftermath of the Sturtian glaciation?

We have now added a description of the Sturtian data on Lines 235-240. *"This sequence of events is remarkably similar to those that occurred in the aftermath of the Neoproterozoic Sturtian glaciation[52]. Following the initial Sturtian deglaciation, a shift to euxinic conditions was followed by a transition to oxygenated conditions. For the majority of the studied locations, there is also a pronounced shift to highly elevated C:P ratios for both organic and reactive P, followed by a return to lower values[52]."*

L241–243: "This may be explained..."
It is very interesting to apply the mechanism proposed by Huybers and Langmuir (2009) for Pleistocene glacial cycles to this context. However, the magnitude of this mechanism is difficult to test even in the Pleistocene because the mechanisms of atmospheric CO2 variations during glacial cycles have not yet been fully understood, although investigations using theoretical models (e.g., Ganopolski and Brovkin, 2017; Köhler, 2023; Kobayashi et al., 2021) are advancing understanding. The reviewer agrees that the pressure change during deglaciation could result in a decline in atmospheric oxygen, but the magnitude and timing of this mechanism are highly uncertain, and this uncertainty could be discussed more carefully by noting it in the manuscript.

We have now highlighted the uncertainty in the proposed mechanism and included the proposed references:

"While there is uncertainty in this depressurization mechanism even for the Pleistocene glaciations, particularly with regard to the overall magnitude and timing of degassing[67], our model results suggest…"

L246: "Specifically, the modelled…"
Same as my comment to L163–172.

This has been addressed above.

Figure 1:
It would be helpful if the glacial period is shown in the figure.

The glacial interval occurs beneath the studied section. We have now noted this in the figure caption.

Figure 2:

Same as my comment to L163–172, but it is questionable that “increased sulfate availability” is directly related to pyrite δ34S.

We have addressed this concern in the major comment above.

Reviewer #3 (Remarks on code availability):

I note that the link for the code provided by the authors did not work.

The link has now been checked and can be found in the supplementary.
https://github.com/lalcott/oneboxScycle.git
