# Supplementary material for: A nutrient control on oxygenation dynamics during Earth’s Great Oxidation Episode
Source: Nat Commun. 2026 Aug 27;17:10294. doi: 10.1038/s41467-026-76597-y (PMC13620156; doi:10.1038/s41467-026-76597-y)
Supplement: Supplementary file 2 [file 41467_2026_76597_MOESM1_ESM.docx]

Supplementary Information for

**A nutrient control on oxygenation dynamics during Earth’s Great Oxidation Episode**

Lewis J. Alcott, Benjamin J. W. Mills, Andrey Bekker, Zidong Peng, Simon W. Poulton

*Corresponding author. Email: lewis.alcott@bristol.ac.uk

**This PDF file includes:**

Supplementary Text

Figs. S1 to S5

Tables S1 to S4

Supplementary Text

Geological and stratigraphic context

The EBA-2 drill-core was drilled near Carltonville in South Africa (Kloof Goldfields Property, Eastern Boundary Area; 26.4700ºS, 27.5883ºE), and comprises well-preserved, low metamorphic grade, early Paleoproterozoic strata of the Transvaal Supergroup. Within the Transvaal sub-basin, the Transvaal Supergroup preserves sediments with ages between ca. 2.65 and 2.05 Ga, and contains the Chuniespoort and Pretoria groups^1^. The studied formations, the Rooihoogte, Timeball Hill and Reitfontein formations, are within the Pretoria Group^1^. Beneath the Rooihoogte Formation, a mid-Transvaal unconformity occurs associated with a Snowball Earth event^2,3^.

Shales of the Rooihoogte Formation are thought to have been deposited relatively proximal to the coastline as part of a fan-delta open to the ocean^4^. The Rooihoogte Formation contains an upper and lower member. Whilst the lower member is not developed in drill-core EBA-2, the upper member includes mudstones and shales coarsening to siltstones^5^. The Timeball Hill Formation is dominated by marine mudstones and shales with thin sandstone beds, suggesting brief intervals of regression in a deltaic setting^4^. The Timeball Hill Formation consists of two upward-coarsening members. The lower member includes interbedded mudstones, siltstones and fine-grained sandstones, with coarser units being interpreted as delta-fed turbidite deposits^6^, while the upper shale member comprises black shales^7^. This overall pattern of sedimentation is thought to reflect progressive continental margin subsidence, providing accommodation for sedimentation^7^. The lower Timeball Hill Member coarsens into the Galsrand Member, a fine- to medium-grained quartz sandstone with oolitic ironstone beds. The Galsrang Member is thought to have been deposited in an oxygenated setting due to the presence of hematite and the highly chemically-weathered nature of the sediments^8^.

The lower Timeball Hill Formation gave ages of 2,309 ± 10 Myr and 2,307 ± 8 Myr based on U-Pb zircon dating of tuff layers^9^, with Re-Os dating of pyrite providing a consistent age of 2,316 ± 7 Myr^10^. The correlative EBA-1 drill-core gave ages of 2,256 ± 6 Myr and 2,266 ± 4 Myr for the upper Timeball Hill Formation^9^, allowing for a hiatus of approximately 40-50 Myr between the lower and upper members.

At the base of the Rooihoogte Formation, a glacial diamictite is commonly present, but it is not developed in EBA-2 drill-core^5^. It is likely missing from the studied section due to erosion at the sequence boundary between the upper and lower Rooihoogte members^11^. This glacial diamictite occurs at other global, low-latitude localities and is considered to signify glacial melting following a possible Snowball Earth glaciation^3,9,12^. The Timeball Hill Formation is overlain by the Rietfontein Member, a glacial diamictite. The upper Timeball Hill Formation has a gradational contact, with the matrix of the overlying diamictite reflecting continued deposition of shale and siltstone^5^. Global correlation of the glacial intervals occurring between ca. 2.46 Ga and 2.22 Ga has been explored previously^3,9,12-15^. We specifically note here that debate concerning global correlations does not affect the mechanistic conclusions and outcomes of this work.

Samples and methods

Samples used in this study are the same as those previously reported^16^, for which Fe speciation, sulphur isotope, and organic carbon data are already available. We performed total dissolutions to obtain redox-sensitive trace element (U, Mo, Re) and major element (P, Al) data, after ashing at 450°C, followed by a HF-HClO_4_-HNO_3_ extraction, with HBO_3_ used to ensure full solubilization of Al (ref. ^16^). Solutions were analyzed by ICP-OES for major elements and ICP-MS for trace elements. Replicate extractions of international sediment standard SGR-1 yielded relative standard deviations (RSDs) of <5% for all elements of interest, with >98% recovery.

Phosphorus phase partitioning was conducted following a revised scheme^17^ developed for ancient sediments (Fig. S1). The method sequentially targets 5 operationally-defined pools, including ferric oxide-bound P (P_Fe1_ and P_Fe2_), authigenic carbonate-fluorapatite (CFA) associated P (P_aut_), crystalline apatite-associated P (P_cryst_), magnetite-associated P (P_mag_), and organic-bound P (P_org_). Phosphorus concentrations for the extracted phases were typically analyzed via spectrophotometry on a Spectonic GENESYS^TM^ 6 at 880 nm, using the molybdate-blue method^18^. The extractants (CDB, CDA, and ammonium oxalate) for the P_Fe_ and P_mag_ fractions interfere with color development using molybdate-blue, and therefore these solutions were analyzed via ICP-OES (for specific details of ICP-OES configuration see ref.^17^). On average, the sum of the extracted phases represented 94% of the total P determined by bulk digests, demonstrating a high recovery. Replicate analyses gave a RSD of ~10% for P_aut_ and P_cryst_, with a value of <13% for P_mag_ and P_org_. P_Fe_ was at very low concentration in our samples (close to the limit of detection), but the relative standard deviation for such analyses is generally ~3% when P_Fe_ is present above trace concentrations^19^, and the very low concentrations of P_Fe_ in our samples do not affect the interpretation of our P phase partitioning data.

Fig. S1. Sequential extraction procedure for phosphorus^17^.

Water column redox reconstruction

The Fe speciation protocol defines an Fe pool that is considered ‘highly reactive’ (Fe_HR_) during sedimentation and diagenesis^20^. Oxic conditions are indicated when Fe_HR_ to total Fe (Fe_T_) ratios are <0.22, while Fe_HR_/Fe_T_ ratios exceeding 0.38 commonly arise from additional water-column precipitation of Fe_HR_ minerals under anoxic conditions^21^. Fe_HR_/Fe_T_ ratios between 0.22 and 0.38 are considered equivocal^21^, and may reflect either oxic or anoxic deposition. Equivocal Fe_HR_/Fe_T_ ratios under anoxic conditions may occur due to masking of the water column precipitation of Fe_HR_ minerals by rapid background sedimentation (e.g., during turbidite deposition) or transfer of non-sulphidized Fe_HR_ minerals to poorly reactive sheet-silicates (Fe_PRS_) during diagenesis and burial metamorphism^16^. As previously explored^16^, the redox interpretation of the EBA-2 drill-core is complicated by a degree of transformation of Fe_HR_ phases to Fe_PRS_. As such, Fe_HR_ data in Figure S2 have been corrected for this transformation, and shown as Fe_HR_^*^ (see refs.^16,22^). For samples deposited from anoxic bottom-waters, the Fe_py_/Fe_HR_ (or Fe_py_/Fe_HR_^*^ in this case) ratio is used to discriminate between ferruginous (Fe_HR_/Fe_T_ > 0.38; Fe_py_/Fe_HR_ < 0.6) and euxinic (Fe_HR_/Fe_T_ > 0.38; Fe_py_/Fe_HR_ > 0.6) water column conditions^23^. The Fe/Al ratio also provides information on water column redox conditions, whereby oxic samples generally fall within an Fe/Al range of 0.55 ± 0.11, and anoxic samples tend to have higher values^24^. Following on from previous assessments of the depositional redox conditions for drill-core EBA-2 (*16*), we consider enrichments in Fe/Al and/or Fe_HR_/Fe_T_ to provide an initial indication of water column anoxia.

To provide a more robust evaluation of local water-column redox conditions, we also utilize Re/U, Re/Mo, and Mo/U ratios^25^, supported by U/Al, Mo/Al, and Re/Al ratios, as well as U and Mo enrichment factors (U_EF_ and Mo_EF_; ref.^26^). Enrichment factors were calculated as:

$\text{Elemen}\text{t}_{\text{EF}}\text{ = }\frac{\text{Element/Al}}{\text{Elemen}\text{t}_{\text{UCC}}\text{/Al}_{\text{UCC}}}$	(1)

where UCC represents upper continental crust^27^.

Uranium and Mo are highly soluble and are thus not generally enriched in oxic sediments ^28^. When anoxia develops at the sediment–water interface, soluble U(VI) is reduced, potentially by ferrous iron^29^, to insoluble U(IV), commonly leading to U enrichment in the sediments^30^. By contrast, the sequestration of high concentrations of Mo requires either the presence of relatively high HS^–^ concentration in the water column, resulting in the formation of thiomolybdates and enrichments in Mo under euxinic conditions^31,32^, or the precipitation of Fe-Mn minerals in the water column, leading to uptake of Mo (but not U or Re) and enrichment in the sediments via a particulate shuttle mechanism (cf. ^26^). A variety of iron minerals may have driven the particulate shuttle during the Archean and Proterozoic. Iron- and Mn-(oxyhydr)oxides are often cited as the principal Mo shuttlers, but within an anoxic ferruginous water column, other phases such as the mixed ferrous-ferric mineral, green rust^33^, or ferrous minerals such as greenalite^34^, may also have precipitated. However, iron silicates such as greenalite are a less efficient shuttle for Mo^35^, suggesting that this phase was not responsible for elevated Mo contents in the sediments.

Rhenium is enriched in sediments just below the sediment–water interface (~1 cm depth), and its enrichment begins under dysoxic conditions^36^. Thus, in combination with Fe speciation data and Fe/Al systematics, RSTE systematics can provide supporting information for redox interpretations, with an additional strength being the potential identification of dysoxic conditions^25^.

These redox proxy parameters are shown for drill-core EBA-2 in Figs. S2 and S3, where Fig. S3 represents an expanded view of the lower part of the succession. To further distinguish between the redox and particulate shuttle pathways, Fig. S4 shows a cross-plot of Mo_EF_ vs. U_EF_, with the particulate shuttle and redox trajectories ^25,47^. As can be seen, euxinia is supported for some samples in accordance with the complimentary redox characteristics derived from Fe speciation systematics. The benefit of this approach is the identification of two samples (top left of fig. S4) demonstrating particulate shutting of Mo as opposed to sulphide driven enrichment.

**Fig. S2. Summary of redox proxies through drill-core EBA-2.** Trends in dominant water-column redox state are based on Fe-S (ref.^16^) and RSTE (this study) systematics. Red lines on U/Al, Mo/Al, and Re/Al plots represent average ratios for upper continental crust^37^. Sulphur isotope data are from ref.^16^, and to emphasize the atmospheric oxygenation history, the Δ^33^S record also includes data from drill-core EBA-1. Red and yellow lines on the Fe_HR_^*^/Fe_T_ plot bound the equivocal zone, where oxic conditions are indicated below the red line, and anoxic conditions are indicated above the yellow line^21^. Red and yellow lines on the Fe_py_/Fe_HR_^*^ plot bound the zone corresponding to anoxic conditions with possible development of euxinia, while values below the red line indicate ferruginous conditions, and those above the yellow line indicate euxinic conditions^38^. Line on the Fe/Al plot indicates the upper limit for identification of anoxic depositional conditions (>0.66; ref.^24^). Intervals A-E correspond to broad atmosphere-ocean redox intervals, as defined by ref.^16^, but we note that higher-frequency fluctuations may have occurred within these broad intervals (cf. ref.^39^). Roo.- Rooihoogte Formation, Riet. – Reitfontein Formation, Bosh. – Boshoek Formation.

**Fig. S3. Summary of redox proxies through the Rooihoogte and lower Timeball Hill formations.** Trends in atmospheric oxygenation and dominant ocean redox state are based on Fe-S (ref.^16^) and RSTE (this study) systematics. Red lines on U/Al, Mo/Al, and Re/Al plots represent average ratios for upper continental crust^37^. Red and yellow lines on the Fe_HR_^*^/Fe_T_ plot bound the equivocal zone, where oxic conditions are indicated below the red line, and anoxic conditions are indicated above the yellow line^21^. Red and yellow lines on the Fe_py_/Fe_HR_^*^ plot bound the zone corresponding to anoxic conditions with possible development of euxinia, while values below the red line indicate ferruginous conditions, and those above the yellow line indicate euxinic conditions^38^. Line on the Fe/Al plot indicates the upper limit for identification of anoxic depositional conditions (>0.66; ref.^24^). Intervals A-D correspond to broad atmosphere-ocean redox intervals, as defined by ref.^16^, but we note that higher-frequency fluctuations may have occurred within these broad intervals (cf. ref.^39^).

**Fig S4. U-EF vs. Mo-EF.** Samples clearly plot within the general patterns of redox covariation for unrestricted marine facies. Symbols reflect redox interpretations as described in the Main Text and Fig. 2. Diagonal lines represent multiples of the Mo:U ratio of present-day seawater (0.3, 1, 3).

Assessing possible P phase partitioning alteration

It is important to acknowledge the potential role diagenesis and metamorphic alteration can play in the interpretation of P phase partitioning data. Iron bound-P and organic P can be readily released to solution during early diagenesis^40-42^. However, any P that remains in the sediment, either through transfer to carbonate fluorapatite or vivianite, or re-adsorption to Fe-(oxyhydr)oxides, is accounted for as part of the overall reactive P pool (P_reac_). Of greater potential impact is the possible phase transfer of P_aut_ to P_cryst_, which has specifically been identified as a factor to consider when utilizing P phase partitioning data for ancient sediments^17,43^, whereby the conversion of P_aut_ to P_cryst_ would lead to an increase in C_org_:P_reac_ ratios. However, even in the highly unlikely scenario that all P_cryst_ was originally present as P_aut_, and thus P_reac_ equates to total P (P_tot_), C_org_:P_tot_ ratios at the Rooihoogte-Timeball Hill boundary still plot above 106:1 (Fig. S4), unequivocally confirming that recycling was occurring across this interval. Therefore, our conclusions are not impacted by possible limited transfer of P_aut_ to P_cryst_.

Fig. S5. Drill-core EBA-2 phosphorus phase partitioning data. Roo.- Rooihoogte Formation, Riet. – Reitfontein Formation.

Biogeochemical modelling

As discussed in the Methods, the biogeochemical model used to generate Figure 4 is a 5-box ocean-atmosphere model containing C-O-P-S cycles^44^ (https://github.com/lalcott/oneboxScycle.git). Parameters and variables used in the model are further described in Tables S1, S2 and S3, and we refer the reader to the original description of the model for additional information^44^. A one-box ocean sulphur cycle model has been included to capture the impact of sulphide on the benthic phosphorus recycling flux. To model the concentration of sulphur within the ocean, we allow for a sulphur input from continental weathering of sulphate evaporites and pyrite. The sulphur flux derived from gypsum is not considered here, as the earliest reported appreciable sulphate evaporite deposits are at ~2.4 Ga^9,14,45^, meaning that if gypsum was extensively buried at this time, it would likely not have been recycled through subduction and collision in time to be weathered and metamorphically degassed during deposition of the studied interval. Reduced sulphur species entering into the ocean-atmosphere system through degassing are considered in the model and are scaled using the present-day input rate^46^. The burial of pyrite depends on the amount of sulphur within the ocean, as well as the ocean oxygen content (Eqn. S80).

The impact of the inclusion of the sulphur cycle is most notably seen in the phosphorus recycling flux calculation (Eqn. S68). Based on the results of our analyses and from previous phosphorus phase partitioning studies of Archean sediments (e.g., ref.^47^), the C:P ratio of organic matter is assumed to be elevated not simply by anoxia as in original iterations of this model (e.g., ref.^44,48^), but particularly by the generation of sulphide via microbial sulphate reduction in sedimentary pore-waters. Therefore, we include a relationship to the rate of pyrite burial in the model, with a scaling factor of ~3000 applied to modelled C:P ratios, to reproduce measured data values.

Table S1. Present-day reservoirs with the corresponding flux equations.

| **Reservoir** | **Eq No.** | **Label** | **Initial Size**  **(moles)** | **Differential Equation** | **Source** |
| --- | --- | --- | --- | --- | --- |
| Proximal Water | 1 | W_P_ | 36x10^12^ | $River_{W}-circ_{PD}$ | [1] |
| Distal Water | 2 | W_D_ | 3600x10^12^ | $circ_{PD}+circ_{DPD}-circ_{DS}$ | [1] |
| Surface Water | 3 | W_S_ | 4,983x10^13^ | $circ_{DPS}+circ_{DS}-circ_{SDP}-Evap$ | [1] |
| Deep Ocean Water | 4 | W_DP_ | 1.3x10^18^ | $circ_{SDP}-circ_{DPS}-circ_{DPD}$ | [1] |
| Proximal Carbon | 5 | C_P_ | 4.5x10^12^ | $PP_{P}-POCmin_{P}-POCbur_{P}-xp_{PD}$ | [1] |
| Distal Carbon | 6 | C_D_ | 243x10^12^ | $xp_{PD}+PP_{D}-POCbur_{D}-POCmin_{D}-xp_{DS}$ | [1] |
| Surface Carbon | 7 | C_S_ | 3,816x10^12^ | $xp_{DS}+PP_{S}-POCmin_{S}-xp_{SDP}$ | [1] |
| Deep Ocean Carbon | 8 | C_DP_ | 5.6x10^16^ | $xp_{SDP}-RespPOC-POCbur_{DP}$ | [1] |
| Proximal Oxygen | 9 | O_P_ | 4.5x10^12^ | $O_{P_{0}}\cdot\boldsymbol{O}_{\boldsymbol{A}}$ | [1] |
| Distal Oxygen | 10 | O_D_ | 243x10^12^ | ${O_{D}}_{0} \cdot\boldsymbol{O}_{\boldsymbol{A}}$ | [1] |
| Surface Oxygen | 11 | O_S_ | 1.615x10^16^ | ${O_{S}}_{O} \cdot\boldsymbol{O}_{\boldsymbol{A}}$ | [1] |
| Deep Ocean Oxygen | 12 | O_DP_ | 2.21x10^17^ | $O_{SDP}-OResp-O_{DPS}-O_{DPD}$ | [1] |
| Atmosphere Oxygen | 13 | O_A_ | 3.7x10^19^ | $POCburT+(2\times mpsb)-AtmosW-genred$ | [2] |
| Proximal SRP | 14 | P_P_ | 9.7x10^9^ | $W_{P}-PPP_{P}+POPmin_{P}-PFe_{P}-Pauth_{P}-SRP_{PD}$ | [1] |
| Distal SRP | 15 | P_D_ | 5x10^12^ | $SRP_{PD}-PPP_{D}+POPmin_{D}-PFe_{D}-Pauth_{D}-SRP_{DS}+SRP_{DPD}$ | [1] |
| Surface SRP | 16 | P_S_ | 47x10^12^ | $SRP_{DS}-PPP_{S}+POPmin_{S}-SRP_{SDP}+SRP_{DPS}$ | [1] |
| Deep Ocean SRP | 17 | P_DP_ | 2,790x10^12^ | $SRP_{SDP}+POPmin_{DP}-PFe_{P}-{Pauth}_{DP}-SRP_{DPS}-SRP_{DPD}$ | [1] |
| Proximal POP | 18 | OP_P_ | 4.3x10^10^ | $PPP_{P}-POPmin_{P}-POPbur_{P}-POP_{PD}$ | [1] |
| Distal POP | 19 | OP_D_ | 2.3x10^12^ | $POP_{PD}+PPP_{D}-POPmin_{D}-POPbur_{D}-POP_{DS}$ | [1] |
| Surface POP | 20 | OP_S_ | 36x10^12^ | $POP_{DS}+PPP_{S}-POPmin_{S}-POP_{SDP}$ | [1] |
| Deep Ocean POP | 21 | OP_DP_ | 530x10^12^ | $POP_{SDP}-POPmin_{DP}-POPbur_{DP}$ | [1] |
| Sulphur Reservoir | 22 | S | 4x10^19^ | $gypw+pyrw+gypdeg+pyrdeg-mgsb-mpsb$ | [5] |

Table S2. Present-day flux values and corresponding equations.

| **Flux** | **Eq No.** | **Label** | **Initial flux**  **(moles/yr)** | **Equation** | **Source** |
| --- | --- | --- | --- | --- | --- |
| River input | 22 | $River_{W}$ | 37x10^12^ | $constant$ | [1] |
| Proximal-Distal water | 23 | $circ_{PD}$ | 37x10^12^ | $constant$ | [1] |
| Low latitude upwelling water | 24 | $circ_{DPD}$ | 3.78x10^14^ | $kDpD\cdot W_{DP}$ | [1] |
| Distal- Surface ocean water | 25 | $circ_{DS}$ | 4.15x10^14^ | $circ_{PD}+circ_{DPD}$ | [1] |
| High latitude upwelling water | 26 | $circ_{DPS}$ | 3.78x10^15^ | $kDpS\cdot W_{DP}$ | [1] |
| High latitude downwelling water | 27 | $circ_{SDP}$ | 4.158x10^15^ | $kSDp\cdot W_{S}$ | [1] |
| Evaporation of surface water | 28 | $Evap$ | 37x10^12^ | $River_{W}$ | [1] |
| Proximal Primary Production OC | 29 | $PP_{P}$ | 3.975x10^13^ | $kPP_{P}\cdot\boldsymbol{P}_{\boldsymbol{P}}\cdot RedCP$ | [1] |
| Proximal OC mineralisation | 30 | $POCmin_{P}$ | 3.277x10^13^ | $kcmin_{P}\cdot\boldsymbol{C}_{\boldsymbol{P}}$ | [1] |
| Proximal OC burial | 31 | $POCbur_{P}$ | 2.3x10^12^ | $kcbur_{P}\cdot PP_{P}$ | [1] |
| Proximal-Distal export prod. | 32 | $xp_{PD}$ | 4.685x10^12^ | $POP_{PD}\cdot RedCP$ | [1] |
| Distal Primary Production OC | 33 | $PP_{D}$ | 5.6x10^14^ | $kPP_{D}\cdot\boldsymbol{P}_{\boldsymbol{D}}\cdot RedCP$ | [1] |
| Distal OC burial | 34 | $POCbur_{D}$ | 1.7x10^12^ | $kcbur_{D}\cdot(xp_{PD}+PP_{D})$ | [1] |
| Distal OC mineralisation | 35 | $POCmin_{D}$ | 5.349x10^14^ | $kcmin_{D}\cdot\boldsymbol{C}_{\boldsymbol{D}}$ | [1] |
| Distal-Surface export prod. | 36 | $xp_{DS}$ | 2.811x10^13^ | $POP_{DS}\cdot RedCP$ | [1] |
| Surface Ocean Primary Production | 37 | $PP_{S}$ | 3.869x10^15^ | $kPP_{S}\cdot\boldsymbol{P}_{\boldsymbol{S}}\cdot RedCP$ | [1]* |
| Surface OC mineralisation | 38 | $POCmin_{S}$ | 3.404x10^15^ | $kcmin_{S}\cdot\boldsymbol{C}_{\boldsymbol{S}}$ | [1]* |
| Surface-Deep export prod. | 39 | $xp_{SDP}$ | 4.931x10^14^ | $kxp_{SDP}\cdot(xp_{DS}+PP_{S})$ | [1]* |
| OC Respiration | 40 | $RespPOC$ | 4.921x10^14^ | $kResp\cdot\boldsymbol{C}_{\boldsymbol{DP}}$ | [1]* |
| Deep Ocean OC burial | 41 | $POCbur_{DP}$ | 1 x10^12^ | $POPburd_{DP}\cdot\frac{\left( CP_{anoxic}\cdot CP_{oxic} \right)}{\left( \left[ \boldsymbol{O}_{\boldsymbol{DP}} \right]\cdot CP_{anoxic} \right)+((1-\left[ \boldsymbol{O}_{\boldsymbol{DP}} \right] )\cdot CP_{oxic})}$ | [1]* |
| Surface-Deep oxygen downwelling | 42 | $O_{SDP}$ | 1.347x10^15^ | $circ_{SDP}\cdot[O_{s}]$ | [1]* |
| Oxygen Respiration | 43 | $OResp$ | 6.403x10^14^ | $kResp\cdot\left( \frac{\boldsymbol{C}_{\boldsymbol{DP}}}{redCO} \right)\cdot\left( \frac{O_{DP}}{\left( kmO_{2}+O_{DP} \right)} \right)$ | [1]* |
| Deep-Surface oxygen upwelling | 44 | $O_{DPS}$ | 6.426x10^14^ | $circ_{DPS}\cdot[O_{DP}]$ | [1] |
| Deep-Distal oxygen upwelling | 45 | $O_{DPD}$ | 6.426x10^13^ | $circ_{DPD}\cdot[O_{DP}]$ | [1] |
| Total OC burial | 46 | $POCburT$ | 5x10^12^ | $POCbur_{P}+POCbur_{D}+POCbur_{DP}$ | [4] |
| Oxidative Weathering | 47 | $AtmosW$ | 5x10^12^ | $kAtmosW\cdot(\sqrt{\boldsymbol{O}_{\boldsymbol{A}}} )$ | [4] |
| Oxygen consumption via reduced gases | 48 | $genred$ | 0 | Varied in Figure 4. | [4] |
| Riverine P input | 49 | $W_{P}$ | 9x10^10^ | Varied in Figure 4. | [1] |
| Proximal Primary Production (P) | 50 | $PPP_{P}$ | 3.75x10^11^ | $\frac{PP_{P}}{RedCP}$ | [1] |
| Proximal POP mineralisation | 51 | $POPmin_{P}$ | 3.216x10^11^ | $\boldsymbol{O}\boldsymbol{P}_{\boldsymbol{P}}\cdot kpmin_{P}$ | [4] |
| Proximal Iron bound P burial | 52 | $PFe_{P}$ | 8.973x10^9^ | $kFeP_{P}\cdot P_{P}\cdot(1-fanoxic_{prox})$ | [4] |
| Proximal Authigenic P burial | 53 | $Pauth_{P}$ | 1.768x10^10^ | $kpmin_{P}\cdot OP_{P}\cdot kCaP_{P}$ | [4] |
| Proximal-Distal SRP transport | 54 | $SRP_{PD}$ | 9.969x10^9^ | $circ_{PD}\cdot[P_{P}]$ | [4] |
| Distal Primary Production (P) | 55 | $PPP_{D}$ | 5.283x10^12^ | $\frac{PP_{D}}{RedCP}$ | [1] |
| Distal POP mineralisation | 56 | $POPmin_{D}$ | 5.055x10^12^ | $\boldsymbol{O}\boldsymbol{P}_{\boldsymbol{P}}\cdot kpmin_{D}$ | [4] |
| Distal Iron bound P burial | 57 | $PFe_{D}$ | 6.762x10^9^ | $kFeP_{D}\cdot P_{D}\cdot(1-fanoxic_{dist})$ | [4] |
| Distal Authigenic P burial | 58 | $Pauth_{D}$ | 1.032x10^10^ | $kCaP_{D}\cdot\boldsymbol{O}_{\boldsymbol{D}}\cdot(1-fanoxic_{dist})$ | [4] |
| Distal-Surface SRP transport | 59 | $SRP_{DS}$ | 5.764x10^11^ | $circ_{DS}\cdot[P_{D}]$ | [1] |
| Deep-Distal SRP transport | 60 | $SRP_{DPD}$ | 8.113x10^11^ | $circ_{DPD}\cdot[P_{DP}]$ | [1] |
| Surface Ocean Primary Production (P) | 61 | $PPP_{S}$ | 3.650x10^13^ | $\frac{PP_{S}}{RedCP}$ | [1]* |
| Surface POP mineralisation | 62 | $POPmin_{S}$ | 3.173x10^13^ | $OP_{S}\cdot kpmin_{S}$ | [4] |
| Surface-Deep SRP transport | 63 | $SRP_{SDP}$ | 3.922x10^12^ | $circ_{SDP}\cdot[P_{S}]$ | [1] |
| Deep-Surface SRP transport | 64 | $SRP_{DPS}$ | 8.113x10^12^ | $circ_{DPS}\cdot[P_{DP}]$ | [1] |
| Deep POP mineralisation | 65 | $POPmin_{DP}$ | 5.028x10^12^ | $OP_{DP}\cdot kpmin_{DP}$ | [4] |
| Deep Iron bound P burial | 66 | $PFe_{DP}$ | 6.75x10^9^ | $kFeP_{DP}\cdot[\boldsymbol{O}_{\boldsymbol{DP}}]$ | [3] |
| Deep Authigenic P burial | 67 | $Pauth_{DP}$ | 1.953x10^10^ | $kCaP_{DP}\cdot POPmin_{DP}\cdot(0.9+\left( 0.1\cdot\left[ \boldsymbol{O}_{\boldsymbol{DP}} \right] \right))$ | [4] |
| C:P ratio for respective box | 68 | $CPratio$ | 250 (oxic) 1100 (anoxic)  Up to 3000 as suggested by the data (euxinic) | $\frac{\left( {\frac{1}{\left( \frac{1-fanoxic_{x}}{CP_{oxic}} \right)+\left( \frac{fanoxic_{x}}{CP_{anoxic}} \right)}} \right)}{S_{Scale}}$ | [1,5] |
| Proximal POP burial | 69 | $POPbur_{P}$ | 9.182x10^9^ | $kcbur_{P}\cdot\frac{PP_{P}}{CPratio_{proximal}}$ | [4] |
| Proximal-Distal POP transport | 70 | $POP_{PD}$ | 4.419x10^10^ | $circ_{PD}\cdot[OP_{P}]$ | [4] |
| Distal POP burial | 71 | $POPbur_{D}$ | 6.800x10^9^ | $kPOPbur_{d}\cdot\frac{PP_{D}+xp_{PD}}{CPratio_{distal}}$ | [4] |
| Distal-Surface POP transport | 72 | $POP_{DS}$ | 2.651x10^11^ | $circ_{DS}\cdot[OP_{D}]$ | [4] |
| Surface-Deep POP transport | 73 | $POP_{SDP}$ | 5.032x10^12^ | $kPOP_{SDP}\cdot\left( \frac{PP_{S}+xp_{DS}}{RedCP} \right)$ | [4] |
| Deep POP burial | 74 | $POPburd_{DP}$ | 4x10^9^ | $kPOPbur_{DP}\cdot\left( \frac{xp_{SDP}}{CP_{oxic}} \right)\cdot(0.9+\left( 0.1*\left[ \boldsymbol{O}_{\boldsymbol{DP}} \right] \right))$ | [4] |
| Gypsum Weathering | 75 | $gypw$ | 0 | Not included in our model runs | [5] |
| Pyrite Weathering | 76 | $pyrw$ | 4.5x10^11^ at present day | $kpyrw\cdot0.6 \cdot pyrw_{O2}$ | [5,6,7] |
| Gypsum Degassing | 77 | $gypdeg$ | 0 | Not included in our model runs |  |
| Pyrite Degassing | 78 | $pyrdeg$ | 0.25x10^12^ at present day | $kpyrdeg\cdot pyrw_{O2}$ | [5,6] |
| Gypsum Burial | 79 | $mgsb$ | 0 | Not included in our model runs | [5] |
| Pyrite Burial | 80 | $mpsb$ | 0.7x10^12^ at present day | $kmpsb\cdot\boldsymbol{POCburT}\cdot log(\boldsymbol{S})$ | [5,6] |
| Oxidative Pyrite Weathering function | 81 | $pyrw_{O2}$ | 1 at present day. Approximation based on Ozaki et al. (2019). | $\frac{\left( {10}^{6}\cdot O2_{PAL} \right)-3}{3497}$ | [5] |
| Sulphur scaling for C:P ratio | 82 | *S_Scale_* | 1 at present day. Allows C:P ratios of up to 3000 in euxinic settings as suggested by the data presented here | $\boldsymbol{mpsb}\cdot3$ | [5] |

OC-Organic carbon, SRP-Soluble-reactive phosphorus, POP-Particulate organic phosphorus

*Flux equation is the same as in the citation, however the present-day value was modified by ref.^56^.

[1] – Slomp and Van Cappellen (2007)^48^

[2] – Bergman et al. (2004)^46^

[3] – Tsandev and Slomp (2009)^49^

[4] – Alcott et al. (2019)^44^

[5] – Newly defined here

[6] – Tostevin and Mills (2020)^50^

[7] - Stüeken et al (2012)^51^

**Table S3. Parameters and Constants.**

| **Parameter/ Constant** | **Constant** | **Value** | **Reference** |
| --- | --- | --- | --- |
| Coastal Upwelling | *kDpD* | 2.908x10^-4^ | ^48^ |
| Open Ocean Upwelling | *kDpS* | 0.0029 | ^48^ |
| Open Ocean Downwelling | *kSDp* | 0.0834 | ^48^ |
| Proximal shelf primary production | *kPP_P_* | 3.75x10^11^ | ^48^ |
| Proximal organic carbon remineralisation | *kcmin_P_* | 3.277x10^13^ | This work |
| Proximal organic carbon burial | *kcbur_P_* | 0.0579 | ^48^ |
| Distal shelf primary production | *kPP_D_* | 5.283x10^12^ | ^48^ |
| Distal organic carbon burial | *kcbur_D_* | 0.003 | ^48^ |
| Distal organic carbon remineralisation | *kcmin_D_* | 5.349x10^14^ | This work |
| Surface ocean primary production | *kPP_S_* | 3.650x10^13^ | ^48^ |
| Surface organic carbon remineralisation | *kcmin_S_* | 3.404x10^15^ | This work |
| Surface to Deep Ocean export production | *kxp_SDP_* | 0.1265 | ^48^ |
| Deep Ocean Respiration | *kResp* | 4.921x10^14^ | ^48^ |
| Deep ocean organic carbon burial | *kcbur_DP_* | 0.0019 | ^48^ |
| Monod constant for respiration | *kmO_2_* | 1x10^-4^ | ^48^ |
| Oxidative Weathering | *kAtmosW* | 5x10^12^ | ^44^ |
| Carbon–phosphorus Redfield ratio | *RedCP* | 106 | ^48^ |
| Proximal iron-bound P burial | *kFeP_P_* | 0.925 | ^44^ |
| Proximal authigenic P burial | *kCaP_P_* | 1.279x10^-12^ | ^44^ |
| Distal iron-bound P burial | *kFeP_D_* | 0.0014 | ^44^ |
| Distal authigenic P burial | *kCaP_D_* | 1.035x10^10^ | ^44^ |
| Deep ocean iron-bound P burial | *kFeP_DP_* | 6.75x10^9^ | ^44^ |
| Deep ocean authigenic P burial | *kCaP_DP_* | 0.0039 | ^44^ |
| Distal organic P burial | *kPOPbur_D_* | 0.003 | ^44^ |
| Surface to deep organic phosphorus export production | *kPOP_SDP_* | 0.1369 | ^44^ |
| Deep ocean organic P burial | *kPOPbur_DP_* | 0.002 | ^44^ |
| Pyrite weathering | *kpyrw* | 4.5x10^11^ | ^52^ |
| Pyrite metamorphic degassing | *kpyrdeg* | 0.25x10^12^ | ^52^ |
| Pyrite burial | *kmpsb* | 0.7x10^12^ | ^52^ |
| Atmospheric oxygen (Present Atmospheric Level) | $O2_{PAL}$ | 1 | ^46^ |

**Table S4. Analytical data, including major and trace element concentrations, C_org_ and P phase partitioning. ND = not determined.**

| Formation | Depth | C_org_ | P_T_ | P_reac_ | P_Fe_ | P_aut_ | P_det_ | P_org_ | Al | Re | U | Mo |
| --- | --- | --- | --- | --- | --- | --- | --- | --- | --- | --- | --- | --- |
|  | (m) | (wt%) | (ppm) | (ppm) | (ppm) | (ppm) | (ppm) | (ppm) | (wt%) | (ppb) | (ppm) | (ppm) |
| Reitfonteindam | 794.00 | 0.05 | 408.6 | 211.2 | 3.5 | 186.9 | 252.1 | 12.9 | 7.5 | 9.0 | 5.7 | 0.6 |
| Reitfonteindam | 794.50 | 0.02 | 419.8 | 262.9 | 4.2 | 240.9 | 200.7 | 12.1 | 5.4 | ND | ND | ND |
| Reitfonteindam | 795.50 | ND | ND | ND | ND | ND | ND | ND | 5.8 | 10.8 | 3.2 | 0.9 |
| Reitfonteindam | 802.00 | 0.06 | 441.6 | 223.4 | 5.4 | 181.9 | 225.5 | 26.3 | 5.9 | 9.8 | 4.2 | 3.0 |
| Reitfonteindam | 802.70 | ND | ND | 293.6 | 0.7 | 270.5 | 196.7 | 13.8 | ND | ND | ND | ND |
| Reitfonteindam | 802.70 | 0.17 | ND | ND | ND | ND | ND | ND | ND | ND | ND | ND |
| Timeball Hill | 803.50 | ND | 317.1 | 117.6 | 2.8 | 65.1 | 80.4 | 45.6 | 10.8 | ND | ND | ND |
| Timeball Hill | 804.10 | ND | 436.7 | 164.9 | 2.8 | 136.5 | 151.5 | 20.0 | 7.9 | ND | ND | ND |
| Timeball Hill | 804.50 | ND | 311.5 | 144.6 | 2.5 | 114.8 | 159.8 | 20.5 | 10.3 | 4.5 | 6.8 | 3.1 |
| Timeball Hill | 806.80 | ND | 802.9 | 331.5 | 6.1 | 278.2 | 435.1 | 33.5 | 7.4 | ND | ND | ND |
| Timeball Hill | 808.00 | 0.30 | ND | ND | ND | ND | ND | ND | 7.0 | 2.0 | 5.5 | 2.9 |
| Timeball Hill | 819.00 | 0.07 | 1167.7 | 550.8 | 10.9 | 507.8 | 674.8 | 19.1 | 6.8 | 12.2 | 3.3 | 1.7 |
| Timeball Hill | 830.00 | 0.10 | 689.0 | 327.7 | 7.1 | 302.8 | 369.6 | 10.5 | 5.6 | 0.3 | 4.0 | 1.3 |
| Timeball Hill | 833.50 | 0.19 | ND | ND | ND | ND | ND | ND | 8.3 | 5.3 | 4.1 | 1.6 |
| Timeball Hill | 849.80 | ND | 481.0 | 237.3 | 0.0 | 224.2 | 284.3 | 5.7 | 9.5 | ND | ND | ND |
| Timeball Hill | 850.00 | 0.40 | ND | ND | ND | ND | ND | ND | 6.9 | 3.2 | 10.9 | 5.0 |
| Timeball Hill | 868.00 | 0.15 | 1658.2 | 788.5 | 1.1 | 735.6 | 694.6 | 39.8 | 3.2 | 9.6 | 3.9 | 1.7 |
| Timeball Hill | 894.00 | 0.29 | 530.9 | 341.9 | 6.2 | 318.8 | 246.0 | 8.2 | 6.8 | 5.3 | 4.7 | 1.1 |
| Timeball Hill | 912.00 | 0.38 | 1730.5 | 738.5 | 14.2 | 683.9 | 1166.8 | 13.4 | 7.2 | 1.4 | 7.5 | 2.2 |
| Timeball Hill | 943.00 | 0.86 | ND | ND | ND | ND | ND | ND | 10.4 | 1.3 | 8.3 | 2.3 |
| Timeball Hill | 943.10 | ND | 613.7 | 333.9 | 2.7 | 313.8 | 339.0 | 7.1 | 11.3 | ND | ND | ND |
| Timeball Hill | 947.50 | 0.86 | ND | ND | ND | ND | ND | ND | 7.6 | 1.2 | 6.1 | 2.3 |
| Timeball Hill | 948.30 | ND | 644.4 | 498.7 | 3.5 | 478.3 | 203.8 | 9.0 | 10.9 | ND | ND | ND |
| Timeball Hill | 993.70 | ND | 293.2 | 168.8 | 2.2 | 141.1 | 177.4 | 20.1 | 9.0 | ND | ND | ND |
| Timeball Hill | 994.00 | 0.66 | ND | ND | ND | ND | ND | ND | 8.6 | ND | ND | ND |
| Timeball Hill | 1002.00 | 0.17 | 506.5 | 267.2 | 3.8 | 242.8 | 305.1 | 12.5 | 6.8 | 2.6 | 3.4 | 1.6 |
| Timeball Hill | 1007.00 | 0.46 | ND | ND | ND | ND | ND | ND | 10.3 | 3.8 | 5.0 | 1.0 |
| Timeball Hill | 1011.00 | ND | 248.1 | 133.6 | 2.0 | 112.9 | 157.8 | 14.8 | 10.6 | ND | ND | ND |
| Timeball Hill | 1012.50 | 0.45 | ND | 161.2 | 4.3 | 137.1 | 169.2 | 13.1 | 11.6 | 7.4 | 5.8 | 0.7 |
| Timeball Hill | 1013.00 | 0.50 | 290.8 | 148.0 | 1.8 | 126.7 | 194.8 | 14.2 | 9.5 | 8.7 | 5.7 | 0.6 |
| Timeball Hill | 1020.00 | 0.18 | ND | ND | ND | ND | ND | ND | 11.1 | 5.3 | 20.5 | 1.0 |
| Timeball Hill | 1022.00 | 0.12 | 286.8 | 111.7 | 1.5 | 79.2 | 179.6 | 25.7 | 9.1 | 16.7 | 13.9 | 10.5 |
| Timeball Hill | 1091.00 | 0.28 | 1102.3 | 457.9 | 14.4 | 405.7 | 579.0 | 20.4 | 11.1 | 0.4 | 8.3 | 3.7 |
| Timeball Hill | 1097.00 | 0.20 | ND | ND | ND | ND | ND | ND | 3.9 | 4.1 | 23.5 | 13.4 |
| Timeball Hill | 1104.00 | 0.14 | 570.8 | 299.4 | 4.2 | 263.8 | 304.6 | 24.1 | 6.6 | ND | ND | ND |
| Timeball Hill | 1112.00 | 0.29 | 330.1 | 213.7 | 3.2 | 182.9 | 97.0 | 22.2 | 12.5 | 0.8 | 8.0 | 5.3 |
| Timeball Hill | 1183.00 | 0.31 | 287.7 | 237.4 | 5.7 | 108.7 | 46.9 | 116.7 | 13.3 | 0.5 | 5.0 | 7.0 |
| Timeball Hill | 1191.00 | 0.29 | 217.3 | 152.5 | 6.2 | 59.9 | 49.3 | 81.4 | 7.7 | 3.1 | 4.7 | 12.3 |
| Timeball Hill | 1202.00 | 0.30 | 210.5 | 154.1 | 5.6 | 61.8 | 34.6 | 81.9 | 12.9 | 0.5 | 4.6 | 1.2 |
| Timeball Hill | 1205.50 | 0.40 | 500.9 | 343.9 | 2.0 | 255.0 | 125.3 | 78.6 | 12.1 | 0.8 | 6.6 | 1.0 |
| Timeball Hill | 1215.50 | 0.30 | 278.1 | 139.2 | 0.3 | 77.4 | 101.6 | 56.1 | 7.4 | 4.4 | 6.0 | 1.9 |
| Timeball Hill | 1224.00 | 0.39 | 219.2 | 167.2 | 2.1 | 83.1 | 51.5 | 75.2 | 9.8 | 0.8 | 6.2 | 1.4 |
| Timeball Hill | 1231.50 | 0.31 | 190.2 | 85.4 | 1.0 | 24.0 | 71.1 | 55.9 | 6.0 | 4.1 | 5.4 | 7.4 |
| Timeball Hill | 1242.50 | 0.22 | 133.6 | 39.0 | 0.3 | 2.8 | 81.0 | 32.1 | 4.7 | 10.0 | 4.4 | 19.1 |
| Timeball Hill | 1253.50 | 0.63 | 276.8 | 155.6 | 1.8 | 64.8 | 75.1 | 82.0 | 9.0 | 1.7 | 6.2 | 6.5 |
| Timeball Hill | 1259.00 | 0.48 | 269.3 | 160.1 | 2.0 | 76.6 | 60.6 | 75.2 | 9.6 | 1.1 | 6.0 | 1.8 |
| Timeball Hill | 1266.00 | 0.50 | 281.2 | 84.8 | 0.4 | 10.6 | 155.3 | 66.5 | 7.7 | 7.1 | 5.9 | 6.4 |
| Timeball Hill | 1275.50 | 0.68 | 239.7 | 121.5 | 1.3 | 32.5 | 54.8 | 82.2 | 10.2 | 2.1 | 6.5 | 2.6 |
| Timeball Hill | 1284.00 | 0.59 | 759.0 | 257.6 | 3.1 | 152.1 | 487.9 | 86.5 | 8.5 | 1.4 | 6.6 | 2.0 |
| Timeball Hill | 1288.30 | 0.76 | 279.7 | 123.1 | 1.5 | 37.5 | 112.6 | 77.8 | 9.1 | 2.5 | 5.7 | 3.8 |
| Timeball Hill | 1296.00 | 0.69 | 352.4 | 116.4 | 2.3 | 41.3 | 189.9 | 64.0 | 7.2 | 3.1 | 6.3 | 4.8 |
| Timeball Hill | 1301.50 | 0.92 | 384.6 | 137.5 | 1.8 | 46.0 | 190.6 | 80.6 | 9.5 | 3.4 | 6.1 | 2.3 |
| Timeball Hill | 1304.00 | 0.69 | 294.5 | 120.8 | 1.4 | 32.3 | 140.3 | 79.1 | 10.9 | 4.3 | 8.7 | 1.7 |
| Timeball Hill | 1312.00 | 0.95 | 592.1 | 152.1 | 3.0 | 43.4 | 424.6 | 92.0 | 9.1 | 2.5 | 7.6 | 3.3 |
| Timeball Hill | 1316.00 | 1.20 | 327.3 | 115.4 | 2.1 | 21.1 | 183.6 | 85.1 | 9.6 | 4.0 | 6.3 | 3.5 |
| Timeball Hill | 1319.00 | 1.11 | 389.7 | 114.8 | 1.8 | 16.1 | 262.8 | 86.8 | 7.4 | 5.3 | 6.8 | 5.3 |
| Timeball Hill | 1324.50 | 0.51 | 285.8 | 190.5 | 2.1 | 83.9 | 70.3 | 98.4 | 10.0 | 1.2 | 7.4 | 1.3 |
| Timeball Hill | 1324.60 | 0.29 | 382.4 | 58.5 | 2.2 | 14.4 | 335.9 | 35.1 | 4.8 | ND | ND | ND |
| Timeball Hill | 1325.50 | 0.63 | 646.9 | 283.4 | 2.7 | 173.9 | 337.0 | 95.3 | 8.0 | 1.7 | 10.4 | 2.7 |
| Timeball Hill | 1327.00 | 0.68 | 179.2 | 77.9 | 1.4 | 10.0 | 104.4 | 59.7 | 7.7 | 0.6 | 5.7 | 1.3 |
| Timeball Hill | 1329.80 | 1.89 | 703.0 | 186.4 | 3.6 | 79.0 | 549.7 | 88.2 | 8.9 | 7.4 | 7.4 | 7.0 |
| Timeball Hill | 1330.00 | 3.49 | 199.7 | 59.0 | 1.9 | 5.9 | 134.2 | 44.3 | 4.4 | 10.8 | 3.9 | 1.0 |
| Timeball Hill | 1331.50 | 1.09 | 433.4 | 88.3 | 2.0 | 11.3 | 349.3 | 62.8 | 5.7 | 11.6 | 2.9 | 1.0 |
| Timeball Hill | 1331.98 | 1.10 | 195.0 | 83.5 | 1.3 | 12.5 | 93.5 | 62.0 | 8.7 | ND | ND | ND |
| Timeball Hill | 1332.01 | 1.23 | 204.3 | 78.6 | 1.2 | 10.9 | 110.4 | 56.9 | 8.8 | ND | ND | ND |
| Timeball Hill | 1332.04 | 1.30 | ND | ND | ND | ND | ND | ND | 5.7 | 5.0 | 3.0 | 0.5 |
| Timeball Hill | 1332.07 | 1.17 | 227.3 | 78.1 | 2.2 | 6.8 | 145.8 | 59.3 | 5.3 | ND | ND | ND |
| Timeball Hill | 1332.10 | 1.14 | 199.1 | 87.9 | 0.8 | 8.4 | 106.7 | 70.4 | 7.7 | ND | ND | ND |
| Timeball Hill | 1332.60 | 1.72 | ND | ND | ND | ND | ND | ND | 8.8 | ND | ND | ND |
| Timeball Hill | 1332.70 | 1.82 | ND | 115.0 | 1.0 | 39.7 | 73.3 | 67.9 | 7.3 | ND | ND | ND |
| Timeball Hill | 1332.80 | 2.81 | ND | 92.2 | 0.9 | 18.7 | 65.8 | 66.6 | 8.5 | 7.5 | 14.0 | 0.6 |
| Timeball Hill | 1333.70 | 4.51 | 365.3 | 182.5 | 2.3 | 92.2 | 173.4 | 78.4 | 4.5 | 9.0 | 13.3 | 1.1 |
| Timeball Hill | 1334.20 | 3.74 | 320.4 | 184.0 | 1.4 | 103.0 | 131.2 | 71.5 | 5.8 | ND | ND | ND |
| Timeball Hill | 1334.30 | 3.30 | ND | ND | ND | ND | ND | ND | 3.6 | ND | ND | ND |
| Timeball Hill | 1334.36 | 2.17 | 164.8 | 52.6 | 1.1 | 6.0 | 124.4 | 40.5 | 3.2 | 16.3 | 5.7 | 2.0 |
| Timeball Hill | 1334.39 | 2.54 | ND | ND | ND | ND | ND | ND | 3.1 | 17.2 | 5.9 | 2.0 |
| Timeball Hill | 1334.43 | 2.50 | ND | ND | ND | ND | ND | ND | 3.7 | 1.3 | 4.4 | 2.0 |
| Timeball Hill | 1334.46 | 2.27 | 189.3 | 71.3 | 1.6 | 4.6 | 95.0 | 59.6 | 3.6 | ND | ND | ND |
| Timeball Hill | 1334.50 | 1.95 | ND | ND | ND | ND | ND | ND | 3.5 | ND | ND | ND |
| Timeball Hill | 1334.60 | 2.92 | ND | ND | ND | ND | ND | ND | 6.7 | 7.8 | 6.8 | 0.7 |
| Rooihoogte | 1335.54 | 1.25 | 162.5 | 195.1 | 24.3 | 50.8 | 79.5 | 119.0 | 1.8 | 8.1 | 5.7 | 11.1 |
| Rooihoogte | 1335.60 | 3.22 | 454.7 | 134.2 | 6.9 | 35.8 | 311.7 | 83.0 | 2.3 | ND | ND | ND |
| Rooihoogte | 1335.66 | 1.25 | ND | ND | ND | ND | ND | ND | 2.2 | ND | ND | ND |
| Rooihoogte | 1335.72 | 3.60 | 386.6 | 192.4 | 7.8 | 106.3 | 199.5 | 71.4 | 1.8 | 8.5 | 5.2 | 0.8 |
| Rooihoogte | 1335.78 | 4.36 | 368.4 | 210.9 | 6.3 | 142.0 | 156.0 | 56.4 | 2.7 | ND | ND | ND |
| Rooihoogte | 1335.84 | 2.37 | ND | ND | ND | ND | ND | ND | 3.1 | ND | ND | ND |
| Rooihoogte | 1335.93 | 3.03 | 183.8 | 47.0 | 1.3 | 4.4 | 137.8 | 36.6 | 2.5 | ND | ND | ND |
| Rooihoogte | 1336.97 | 2.59 | 213.1 | 48.6 | 1.2 | 3.8 | 191.9 | 38.2 | 3.6 | 9.2 | 5.1 | 0.8 |
| Rooihoogte | 1336.03 | 2.79 | ND | ND | ND | ND | ND | ND | 4.1 | ND | ND | ND |
| Rooihoogte | 1336.09 | 2.24 | 226.2 | 41.6 | 1.5 | 5.5 | 213.2 | 29.7 | 2.6 | ND | ND | ND |
| Rooihoogte | 1336.14 | 2.07 | 1207.0 | 69.0 | 3.9 | 16.1 | 1165.7 | 31.9 | 2.3 | ND | ND | ND |
| Rooihoogte | 1336.19 | 2.19 | ND | ND | ND | ND | ND | ND | 2.4 | 8.7 | 2.4 | 0.4 |
| Rooihoogte | 1336.23 | 2.87 | 225.3 | 51.9 | 2.3 | 2.9 | 188.4 | 40.9 | 2.2 | ND | ND | ND |
| Rooihoogte | 1336.27 | 2.49 | 746.0 | 66.2 | 2.8 | 10.4 | 679.2 | 40.9 | 2.3 | 13.6 | 3.0 | 0.9 |
| Rooihoogte | 1336.29 | 2.65 | ND | ND | ND | ND | ND | ND | 2.6 | 17.4 | 3.2 | 0.9 |
| Rooihoogte | 1336.33 | 1.97 | 352.6 | 46.0 | 1.3 | 5.0 | 304.8 | 32.4 | 3.0 | ND | ND | ND |
| Rooihoogte | 1336.35 | 1.05 | 238.8 | 61.0 | 5.7 | 6.6 | 207.6 | 44.4 | 2.0 | 8.5 | 6.0 | 2.4 |
| Rooihoogte | 1336.39 | 2.93 | ND | ND | ND | ND | ND | ND | 5.5 | 5.9 | 5.6 | 4.0 |
| Rooihoogte | 1336.44 | 2.95 | 511.4 | 228.7 | 1.6 | 172.6 | 282.3 | 45.7 | 3.4 | 7.8 | 2.4 | 111.7 |
| Rooihoogte | 1336.47 | 2.01 | ND | ND | ND | ND | ND | ND | 1.9 | 6.4 | 6.2 | 12.3 |
| Rooihoogte | 1336.50 | 2.53 | 361.9 | 110.5 | 6.2 | 17.3 | 280.5 | 79.3 | 4.7 | 10.6 | 1.8 | 139.3 |
| Rooihoogte | 1337.00 | 0.41 | 303.8 | 169.3 | 0.5 | 123.6 | 125.2 | 38.5 | 11.6 | 1.2 | 4.0 | 0.4 |
| Rooihoogte | 1337.20 | 0.78 | 683.1 | 53.7 | 1.7 | 7.4 | 643.9 | 28.7 | 3.5 | ND | ND | ND |
| Rooihoogte | 1337.24 | 0.64 | 397.1 | 64.2 | 2.0 | 10.9 | 312.7 | 40.1 | 6.2 | 1.3 | 4.0 | 0.4 |
| Rooihoogte | 1337.27 | 0.63 | 207.6 | 104.1 | 2.1 | 18.7 | 75.8 | 75.3 | 6.7 | ND | ND | ND |
| Rooihoogte | 1337.32 | 0.57 | 359.5 | 194.1 | 2.7 | 85.4 | 121.6 | 93.6 | 8.5 | ND | ND | ND |
| Rooihoogte | 1337.38 | 0.87 | 272.7 | 82.4 | 2.2 | 9.5 | 175.3 | 60.8 | 5.0 | ND | ND | ND |
| Rooihoogte | 1337.43 | 1.37 | 173.3 | 99.1 | 2.0 | 45.9 | 64.4 | 45.6 | 5.2 | ND | ND | ND |
| Rooihoogte | 1337.50 | 1.01 | 110.3 | 42.8 | 0.6 | 4.8 | 62.4 | 33.6 | 3.0 | 1.4 | 16.5 | 0.8 |
| Rooihoogte | 1337.70 | 2.39 | 262.8 | 235.8 | 109.5 | 80.9 | 129.6 | 40.5 | 4.2 | 0.8 | 14.8 | 0.4 |
| Rooihoogte | 1337.70 | 0.56 | ND | ND | ND | ND | ND | ND | 8.3 | ND | ND | ND |
| Rooihoogte | 1338.07 | 1.43 | 248.4 | 76.2 | 26.1 | 6.6 | 4.8 | 43.0 | 2.2 | ND | ND | ND |
| Rooihoogte | 1338.10 | 2.72 | ND | ND | ND | ND | ND | ND | 1.4 | ND | ND | ND |
| Rooihoogte | 1338.13 | 3.01 | ND | ND | ND | ND | ND | ND | 4.9 | 15.4 | 17.1 | 55.5 |
| Rooihoogte | 1338.17 | 2.70 | 223.6 | 164.1 | 3.1 | 78.3 | 29.1 | 80.5 | 1.9 | ND | ND | ND |
| Rooihoogte | 1338.20 | 1.91 | ND | ND | ND | ND | ND | ND | 1.8 | 20.5 | 31.7 | 17.4 |
| Rooihoogte | 1338.24 | 3.31 | 279.2 | 232.3 | 51.7 | 127.5 | 92.6 | 47.8 | 4.3 | 7.8 | 14.5 | 8.7 |
| Rooihoogte | 1338.30 | 1.05 | 120.3 | 56.5 | 1.1 | 9.7 | 3.3 | 45.2 | 1.9 | 12.2 | 10.3 | 14.3 |
| Rooihoogte | 1338.50 | 0.44 | ND | ND | ND | ND | ND | ND | 14.7 | 0.7 | 31.5 | 0.6 |
| Rooihoogte | 1338.70 | 0.56 | ND | ND | ND | ND | ND | ND | 8.6 | ND | ND | ND |
| Rooihoogte | 1339.00 | 0.41 | 795.7 | 372.8 | 18.9 | 241.4 | 429.2 | 95.3 | 15.6 | ND | ND | ND |
| Rooihoogte | 1340.50 | 0.26 | ND | ND | ND | ND | ND | ND | 9.7 | 0.3 | 0.9 | 0.6 |
| Rooihoogte | 1343.00 | 0.34 | 2177.5 | 723.8 | 1.4 | 613.3 | 1514.4 | 78.3 | 5.1 | 6.0 | 3.8 | 3.5 |
| Rooihoogte | 1344.50 | 0.53 | 254.3 | 149.4 | 0.7 | 50.3 | 98.8 | 89.7 | 8.7 | 2.5 | 4.6 | 1.8 |
| Rooihoogte | 1345.50 | 0.56 | 498.9 | 300.7 | 2.3 | 185.4 | 181.8 | 101.8 | 9.3 | 1.9 | 3.4 | 2.1 |
| Rooihoogte | 1346.00 | 0.39 | 398.3 | 284.2 | 3.4 | 135.8 | 106.6 | 131.8 | 10.8 | 0.6 | 11.8 | 1.6 |
| Rooihoogte | 1346.10 | 0.32 | 685.0 | 383.9 | 18.0 | 255.2 | 341.7 | 94.9 | 7.9 | ND | ND | ND |
| Rooihoogte | 1346.12 | 0.22 | 332.2 | 435.1 | 57.5 | 102.6 | 103.7 | 269.2 | 3.2 | ND | ND | ND |
| Rooihoogte | 1346.14 | 0.32 | ND | 731.5 | 2.9 | 249.3 | 156.1 | 472.3 | 4.7 | ND | ND | ND |
| Rooihoogte | 1346.16 | 0.81 | ND | 406.8 | 4.9 | 247.6 | 132.1 | 140.2 | 11.9 | ND | ND | ND |
| Rooihoogte | 1346.20 | 0.34 | ND | ND | ND | ND | ND | ND | 9.4 | 0.5 | 4.4 | 2.8 |

**References**

1	Moore, J. M., Tsikos, H. & Polteau, S. Deconstructing the Transvaal Supergroup, South Africa: implications for Palaeoproterozoic palaeoclimate models. *Journal of African Earth Sciences* **33**, 437-444 (2001).

2	Eriksson, P. G., Hattingh, P. J. & Altermann, W. An overview of the geology of the Transvaal Sequence and Bushveld Complex, South Africa. *Mineralium Deposita* **30**, 98-111 (1995).

3	Gumsley, A. P. *et al.* Timing and tempo of the Great Oxidation Event. *PNAS* **114**, 1811-1816 (2017). <https://doi.org/https://doi.org/10.1073/pnas.1608824114>

4	Catuneanu, O. & Eriksson, P. G. Sequence stratigraphy of the Precambrian Rooihoogte–Timeball Hill rift succession, Transvaal Basin, South Africa. *Sedimentary Geology* **147**, 71-88 (2002).

5	Coetzee, L. L. *Genetic Stratigraphy of the Paleoproterozoic Pretoria Group in the Western Transvaal*, Rand Afrikaans Univ., (2001).

6	Eriksson, P. G. & Reczko, B. F. F. The sedimentary and tectonic setting of the Transvaal Supergroup floor rocks to the Bushveld Complex. *Journal of African Earth Sciences* **21**, 487-504 (1995).

7	Catuneanu, O. & Eriksson, P. G. The sequence stratigraphic concept and the Precambrian rock record: an example from the 2.3–2.1 Ga Pretoria Group, Kaapvaal craton. *Precambrian Research* **97**, 215-251 (1999).

8	Coetzee, L. L., Beukes, N. J., Gutzmer, J. & Kakegawa, T. Links of organic carbon cycling and burial to depositional depth gradients and establishment of a snowball Earth at 2.3 Ga. Evidence from the Timeball Hill Formation, Transvaal Supergroup, South Africa. *S Afr J Geol* **109**, 109-122 (2006).

9	Rasmussen, B., Bekker, A. & Fletcher, I., R. Correlation of Paleoproterozoic glaciations based on U-Pb zircon ages for tuff beds in the Transvaal and Huronian Supergroups. *Earth and Planetary Science Letters* **382**, 173-180 (2013).

10	Hannah, J. L., Bekker, A., Stein, H. J., Markey, R. J. & Holland, H. D. Primitive Os and 2316 Ma age for marine shale: implications for Paleoproterozoic glacial events and the rise of atmospheric oxygen. *Earth and Planetary Science Letters* **225**, 43-52 (2004).

11	Luo, G. *et al.* Rapid oxygenation of Earth’s atmosphere 2.33 billion years ago. *Science Advances* **2** (2016).

12	Bekker, A., Krapež, B. & Karhu, J. A. Correlation of the stratigraphic cover of the Pilbara and Kaapvaal cratons recording the lead up to Paleoproterozoic Icehouse and the GOE. *Earth-Science Reviews* **211**, 103389 (2020).

13	Bekker, A. & Kaufman, A., J. Oxidative forcing of global climate change: a biogeochemical record across the oldest Paleoproterozoic ice age in North America. *Earth Planet. Sci. Lett.* **258**, 486-499 (2007).

14	Rasmussen, B., Zi, J. & Bekker, A. New U-Pb zircon tuff ages and revised stratigraphic correlations in the Superior craton during the Great Oxidation Episode. *Earth and Planetary Science Letters* **640** (2024).

15	Hoffman, P. F. The Great Oxidation and a Siderian snowball Earth: MIF-S based correlation of Paleoproterozoic glacial epochs. *Chemical Geology* **362**, 143-156 (2013).

16	Poulton, S. W. *et al.* A 200-million-year delay in permanent atmospheric oxygenation. *Nature* **592**, 232-236 (2021).

17	Thompson, J. *et al.* Development of a modified SEDEX phosphorus speciation method for ancient rocks and modern iron-rich sediments. *Chemical Geology* **524**, 383-393 (2019).

18	Strickland, J. D. H. & Parsons, T. R. A practical handbook of seawater analysis. *(2nd edition), Fisheries Research Board of Canada, Ottawa, Bulletin* **167**, 45-64 (1972).

19	Thompson, J. *et al.* Development of a modified SEDEX phosphorus speciation method for ancient rocks and modern iron-rich sediments. *Chemical Geology* **524**, 383-393 (2019).

20	Raiswell, R. & Canfield, D., E. Sources of iron for pyrite formation in marine sediments. *Am. J. Sci.* **298**, 219-245 (1998).

21	Poulton, S. W. & Canfield, D. E. Ferruginous Conditions: A Dominant Feature of the Ocean through Earth's History. *Elements* **7**, 107-112 (2011).

22	Ostrander, C. M. *et al.* Onset of coupled atmosphere–ocean oxygenation 2.3 billion years ago. *Nature* **631**, 335-339 (2024).

23	Poulton, S. W. in *Geochemical Tracers in Earth System Science*	(eds T Lyons, W., A. Turchyn, & C Reinhard, T.) Ch. The Iron Speciation Paleoredox Proxy, 1-20 (Cambridge University Press, 2021).

24	Clarkson, M., O., Poulton, S., W, Guilbaud, R. & Wood, R., A. Assessing the utility of Fe/Al and Fe-speciation to record water column redox conditions in carbonate-rich sediments. *Chemical Geology* **382**, 111-122 (2014).

25	Li, S., Wignall, P. B. & Poulton, S. W. Co-application of rhenium, vanadium, uranium and molybdenum as paleo-redox proxies: Insight from modern and ancient environments. *Chemical Geology* **674** (2025).

26	Tribovillard, N., Algeo, T. J., Baudin, F. & Riboulleau, A. Analysis of marine environmental conditions based on molybdenum-uranium covariation—Applications to Mesozoic paleoceanography. *Chemical Geology* **324-325**, 46-58 (2012).

27	Taylor, S. R. & McLennan, S. M. *The Continental Crust: Its Composition and Evolution*. (Blackwell, 1985).

28	Morford, J. L., Martin, W. R. & Carney, C. M. Uranium diagenesis in sediments underlying bottom waters with high oxygen content. *Geochimica et Cosmochimica Acta* **73**, 2920-2937 (2009).

29	Cole, D. B. *et al.* Uranium Isotope Fractionation in Non-sulfidic Anoxic Settings and the Global Uranium Isotope Mass Balance. *Global Biogeochemical Cycles* **34**, e2020GB006649 (2020).

30	Anderson, R. F., Fleisher, M. Q. & LeHuray, A. P. Concentration, oxidation state, and particulate flux of uranium in the Black Sea. *Geochimica et Cosmochimica Acta* **53**, 2215-2224 (1989).

31	Helz, G. R. *et al.* Mechanism of molybdenum removal from the sea and its concentration in black shales: EXAFS evidence. *Geochimica et Cosmochimica Acta* **60**, 3631-3642 (1996).

32	Phillips, R. & Xu, J. A critical review of molybdenum sequestration mechanisms under euxinic conditions: Implications for the precision of molybdenum paleoredox proxies. *Earth-Science Reviews* **221** (2021).

33	Zegeye, A. *et al.* Green rust formation controls nutrient availability in a ferruginous water column. *Geology* **40**, 599-602 (2012).

34	Hinz, I. L., Nums, C., Theuer, S., Templeton, A. S. & Johnson, J. E. Ferric iron triggers greenalite formation in simulated Archean seawater. *Geology* **49**, 905-910 (2021).

35	Tostevin, R. & Ahmed, I. A. M. Micronutrient availability in Precambrian oceans controlled by greenalite formation. *Nature Geoscience* **16**, 1188-1193 (2023).

36	Crusius, J., Calvert, S., Pedersen, T. & Sage, D. Rhenium and molybdenum enrichments in sediments as indicators of oxic, suboxic and sulfidic conditions of deposition. *Earth and Planetary Science Letters* **145**, 65-78 (1996).

37	McLennan, S. M. Relationships between the trace element composition of sedimentary rocks and upper continental crust. *Geochemistry, Geophysics, Geosystems* **2** (2001).

38	Poulton, S., W. in *Geochemical Tracers in Earth System Science*	(eds T Lyons, W., A. Turchyn, & C Reinhard, T.) Ch. The Iron Speciation Paleoredox Proxy, 1-20 (Cambridge University Press, 2021).

39	Uveges, B. T., Izon, G., Ono, S., Beukes, N. J. & Summons, R. E. Reconciling discrepant minor sulfur isotope records of the Great Oxidation Event. *Nature Communications* **14** (2023).

40	Ruttenberg, K. C. & Berner R, A. Authigenic apatite formation and burial in sediments from non-upwelling, continental margin environments. *Geochimica et Cosmochimica Acta* **57**, 991-1007 (1993).

41	Ingall, E. D., Bustin, R. M. & Van Cappellen, P. Influence of water column anoxia on the burial and preservation of carbon and phosphorus in marine shales. *Geochimica et Cosmochimica Acta* **57**, 303-316 (1993).

42	Slomp, C. P., Epping, E. H. G., Helder, W. & Van Raaphorst, W. A key role for iron-bound phosphorus in authigenic apatite formation in North Atlantic continental platform sediments. *Journal of Marine Research* **54**, 1179-1205 (1996).

43	Creveling, J. R. *et al.* Phosphorus sources for phosphatic Cambrian carbonates. *GSA Bulletin* **126**, 145-163 (2014).

44	Alcott, L. J., Mills, B., J. W. & Poulton, S. W. Stepwise Earth oxygenation is an inherent property of global biogeochemical cycling. *Science* **366**, 1333-1337 (2019).

45	Yudoskaya, M. A. *et al.* in *Metals, Minerals, and Society* Vol. 21 (eds A. M Arribas & J. L Mauk) (Society of Economic Geologists, 2018).

46	Bergmann, N. M., Lenton, T., M & Watson, A. J. COPSE: a new model of biogeochemical cycling over Phanerozoic time. *Am. J. Sci.* **304**, 397-437 (2004).

47	Alcott, L. J., Mills, B. J. W., Bekker, A. & Poulton, S. W. Earth’s Great Oxidation Event facilitated by the rise of sedimentary phosphorus recycling. *Nature Geoscience* **15**, 210-215 (2022).

48	Slomp, C. P. & Van Cappellen, P. The global marine phosphorus cycle: sensitivity to oceanic circulation. *Biogeosciences* **4**, 155-171 (2007).

49	Tsandev, I. & Slomp, C. P. Modeling phosphorus cycling and carbon burial during Cretaceous Oceanic Anoxic Events. *Earth and Planetary Science Letters* **286**, 71-79 (2009).

50	Tostevin, R. & Mills, B. J. W. Reconciling proxy records and models of Earth's oxygenation during the Neoproterozoic and Palaeozoic. *Interface Foxus* **10** (2020).

51	Stüeken, E. E., Catling, D. C. & Buick, R. Contributions to late Archaean sulphur cycling by life on land. *Nature Geoscience* **5**, 722-725 (2012).

52	Shields, G., A. & Mills, B., J, W. Tectonic controls on the long-term carbon isotope mass balance. *PNAS* **114**, 4318-4323 (2017).
